# Genomic characterization of three marine fungi, including *Emericellopsis atlantica* sp. nov. with signatures of a generalist lifestyle and marine biomass degradation

**DOI:** 10.1186/s43008-021-00072-0

**Published:** 2021-08-09

**Authors:** Ole Christian Hagestad, Lingwei Hou, Jeanette H. Andersen, Espen H. Hansen, Bjørn Altermark, Chun Li, Eric Kuhnert, Russell J. Cox, Pedro W. Crous, Joseph W. Spatafora, Kathleen Lail, Mojgan Amirebrahimi, Anna Lipzen, Jasmyn Pangilinan, William Andreopoulos, Richard D. Hayes, Vivian Ng, Igor V. Grigoriev, Stephen A. Jackson, Thomas D. S. Sutton, Alan D. W. Dobson, Teppo Rämä

**Affiliations:** 1 Marbio, The Norwegian College of Fishery Science, Department at Faculty of Biosciences, Fisheries and Economics, UiT The Arctic University of Norway, Tromsø, Norway; 2 Westerdijk Fungal Biodiversity Institute, Uppsalalaan 8, 3584CT Utrecht, Netherlands; 3 The Norwegian Structural Biology Centre (NorStruct), Department of Chemistry, Faculty of Science and Technology, UiT the Arctic University of Norway, Tromsø, Norway; 4 Institute of Organic Chemistry and BMWZ, Leibniz Universität Hannover, Hanover, Germany; 5 Department of Botany and Plant Pathology, Oregon State University, Corvallis, USA; 6 US Department of Energy Joint Genome Institute, Lawrence Berkeley National Laboratory, Berkeley, CA 94720 USA; 7 Department of Plant and Microbial Biology, University of California Berkeley, Berkeley, CA 94720 USA; 8 School of Microbiology, University College Cork, Cork, Ireland; 9 MaREI Centre, Environmental Research Institute, University College Cork, Cork, Ireland; 10 APC Microbiome Ireland, Cork, Ireland

**Keywords:** Bioprospecting, Genome mining, Illumina, Lignocellulolytic enzymes, Physiology, Taxonomy, 1 new taxon

## Abstract

Marine fungi remain poorly covered in global genome sequencing campaigns; the 1000 fungal genomes (1KFG) project attempts to shed light on the diversity, ecology and potential industrial use of overlooked and poorly resolved fungal taxa. This study characterizes the genomes of three marine fungi: *Emericellopsis* sp. TS7, wood-associated *Amylocarpus encephaloides* and algae-associated *Calycina marina.* These species were genome sequenced to study their genomic features, biosynthetic potential and phylogenetic placement using multilocus data. *Amylocarpus encephaloides* and *C. marina* were placed in the *Helotiaceae* and *Pezizellaceae (Helotiales)*, respectively, based on a 15-gene phylogenetic analysis. These two genomes had fewer biosynthetic gene clusters (BGCs) and carbohydrate active enzymes (CAZymes) than *Emericellopsis* sp. TS7 isolate. *Emericellopsis* sp. TS7 (*Hypocreales*, *Ascomycota*) was isolated from the sponge *Stelletta normani*. A six-gene phylogenetic analysis placed the isolate in the marine *Emericellopsis* clade and morphological examination confirmed that the isolate represents a new species, which is described here as *E. atlantica*. Analysis of its CAZyme repertoire and a culturing experiment on three marine and one terrestrial substrates indicated that *E. atlantica* is a psychrotrophic generalist fungus that is able to degrade several types of marine biomass. FungiSMASH analysis revealed the presence of 35 BGCs including, eight non-ribosomal peptide synthases (NRPSs), six NRPS-like, six polyketide synthases, nine terpenes and six hybrid, mixed or other clusters. Of these BGCs, only five were homologous with characterized BGCs. The presence of unknown BGCs sets and large CAZyme repertoire set stage for further investigations of *E. atlantica*. The *Pezizellaceae* genome and the genome of the monotypic *Amylocarpus* genus represent the first published genomes of filamentous fungi that are restricted in their occurrence to the marine habitat and form thus a valuable resource for the community that can be used in studying ecological adaptions of fungi using comparative genomics.

**Supplementary Information:**

The online version contains supplementary material available at 10.1186/s43008-021-00072-0.

## INTRODUCTION

The first genome of a fungus, *Saccharomyces cerevisiae*, was sequenced in 1996 (Goffeau et al. 1996). Subsequent developments in technology have made sequencing much more affordable, and the number of fungal genome and transcriptome sequencing projects has increased exponentially resulting in 1886 genomes being available in 2020 (Grigoriev et al. 2014; Sharma 2015; NCBI 2021). Most of the early sequencing efforts were focused on terrestrial ecologically or economically significant fungi, crop-pathogens, or fungi related to human health (Sharma 2015). Despite the effort so far, one issue in comparative genomics is the lack of available genomic data and proper taxonomic representation of the known taxa (Naranjo-Ortiz and Gabaldón 2019; Lücking et al. 2020). This is especially noticeable among marine fungi, where few genomes are available compared to terrestrial fungi. The 1000 fungal genomes (1KFG) project wants to address these issues and answer questions regarding ecologically and taxonomically overlooked fungi like marine fungi in poorly resolved taxa, such as *Helotiales* (*Leotiomycetes*). By making their genomes publicly available, 1KFG contributes to better elucidate the general features of marine fungi (Grigoriev et al. 2011; Grigoriev et al. 2014).

The marine environment is vastly different from the terrestrial environment, leading to distinct adaptations of the organisms living there. Such adaptations may be unique enzymes that withstand low or high temperatures, pressure or salt concentrations, and potent signaling molecules and sensitive receptors, specific pigments, and other unique metabolites (Van Noort et al. 2013; Kis-Papo et al. 2014; Rédou et al. 2015; Oey 2016; Fouillaud et al. 2017; Huang et al. 2017; Trincone 2018). There are many substrates available in the marine environment that are different compared to terrestrial substrates. Such substrates include polysaccharides such as laminarin, carrageenan, fucoidan, alginate, ulvan, galactans, porphyrin, agarose and chitin that do not occur in terrestrial sources or have different modifications such as sulfation (Barbosa et al. 2019). Fungal enzymes utilizing specific marine polysaccharides, such as glycoside hydrolase family 29 (GH29) linked to the degradation of algal fucoidan, GH107 linked to sulfated fucans, GH78 and GH105 linked to ulvan and GH18 and GH82 linked to carrageenan, are of interest for industrial processing. These enzymes make sugars bioavailable in feed for aquaculture and agriculture, usable in the production of specific polysaccharides for pharmaceutical purposes or as a carbon source for bioenergy production. Marine microorganisms also communicate with each other and protect themselves using secondary metabolites. Because the water dilutes any secreted molecules, the secondary metabolites have to be potent and they are therefore of special pharmaceutical interest as potential drugs (Berteau et al. 2002; Haefner 2003; Michel et al. 2006; Collén et al. 2014; Vickers et al. 2018; Reisky et al. 2019; Carroll et al. 2020; Dobrinčić et al. 2020).

Some of the fungi frequently observed in the marine environment include *Acremonium*-like fungi that are a polyphyletic assembly of mostly indistinct, hyaline, simple, asexual fungi. These fungi are isolated from macroalgae, invertebrates and sediments (Zuccaro et al. 2008; Duc et al. 2009; Loque et al. 2010; Paz et al. 2010; Mouton et al. 2012; Zhang et al. 2013; Rédou et al. 2015; Zhang et al. 2015; Lee et al. 2019). Binomially named *Acremonium* fungi are found within *Glomerellales*, *Hypocreales*, *Sordariales*, *Cephalothecales* (*Cephalothecaceae)* and *Leotiomycetes* showing how *Acremonium* is used collectively on phylogenetically distinct, but often morphologically indistinct fungi (Summerbell et al. 2011). Many of these fungi have close sequence similarity to sexual reproductive morph of described species and likely represent the asexual morphs of these species (Summerbell et al. 2011). Some of the *Acremonium*-like taxa within the *Emericellopsis* clade are marine, specifically those closely related to *E. maritima* and *A. fuci,* whereas terrestrial isolates form a distinct clade (Zuccaro et al. 2004). Alkali-tolerant soda soil fungi seem to have derived from the marine lineage and are nested in their own subclade within the marine clade (Grum-Grzhimaylo et al. 2013). This concept of three ecological clades is challenged by recent research based on nuclear ribosomal DNA (nrDNA) ITS1–5.8S-ITS2 region (ITS) and β-tubulin (*tub2*) phylogeny and should be retested with multilocus gene phylogenies when new species are described (Gonçalves et al. 2020). Despite frequent phylogenetic studies and descriptions of new species, relatively few *Acremonium*-like fungi have available genome sequences. For *Emericellopsis*, there are no reference genomes available (Grigoriev et al. 2014; NCBI Resource Coordinators 2018). From chemical studies, it is known that species within the genus of *Acremonium* and *Emericellopsis* can produce a range of known bioactive metabolites (Argoudelis et al. 1974; Rogozhin et al. 2018; Hsiao et al. 2020). Despite evidence of secondary metabolite production, our understanding of the full biosynthetic potential of *Emericellopsis* species remains limited.

*Calycina marina* is a non-lichenized discomycetous fungus that is exclusively found on decaying seaweeds and has been collected all over the northern Europe (Baral and Rämä 2015; GBIF Secretariat 2021). *Calycina marina* is unique in both habitat, substrate and morphology compared to its closest relatives in *Calycina* that are terrestrial species (Baral and Rämä 2015). It is also peculiar in the sense that it is one of the few marine discomycetes compared to the terrestrial environment with hundreds of discomycetous species. *Amylocarpus encephaloides* is another strictly marine fungus that occurs on wood in the tidal zone (Prasannarai and Sridhar 2004). The fungus has a unique way of degrading wood that is similar to brown rot, but distinct from it, which may involve industrially interesting CAZymes (Prasannarai and Sridhar 2004). The fungus has been reported from in the Atlantic, Pacific and Indian Ocean (Prasannarai and Sridhar 2004; GBIF Secretariat 2021).

Here, we provide a thorough taxonomic and genomic description of the first fully sequenced *Emericellopsis* species. To further contribute to the knowledge of marine fungi, we include a brief description of the genomes of two marine fungi, *Calycina marina* and *Amylocarpus encephaloides* (*Helotiales*, *Ascomycota*), and resolve their phylogeny based on multilocus data extracted from genome sequences.

## MATERIALS AND METHODS

In this manuscript we adhere to italicizing Latin names of organisms and higher order taxonomic ranks as discussed in Thines et al. (2020). Several of the methods used have previously been published and are only briefly described here.

### Sampling and isolate information

The isolation method of the isolate TS7 was previously described in Batista-García et al. (2017). *Emericellopsis* sp. TS7 (Class *Sordariomycetes*, Order *Hypocreales*, Family *Hypocreales incertae sedis*) was obtained from the sponge *Stelletta normani* (Class *Demospongiae*, Order *Astrophorida*, Family *Ancorinidae*) collected on 16th June 2010 from 1350 m depth in the Atlantic Ocean (54.0613° N, 12.5518° W), off the west coast of Ireland using a remote operated vehicle *Holland I* on board the *R.V. Explorer* (Kennedy et al. 2014). Briefly, 1 mL of the macerated sponge material was serially diluted and 100 μL of each dilution was inoculated on agar plates with either malt extract agar-artificial seawater (ASW) or potato dextrose agar-ASW (DIFCO). Axenic cultures were obtained after two passages from the primary isolation. The fungus is accessible in the fungal collection of the School of Microbiology at University College Cork, under accession code TS7, and the Westerdijk Fungal Biodiversity Institute (CBS-KNAW) under the accession CBS 147198. *Emericellopsis* sp. TS7 was selected for full genome sequencing in the 1KFG project due to the lack of sequenced *Emericellopsis* species, its marine origin, promising antibacterial activity against gram-negative bacteria in initial bioactivity testing and as a putative novel species (Jackson et al. 2016).

Isolation of *C. marina* TRa3180A (Class *Leotiomycetes*, Order *Helotiales*, Family *Pezizellaceae*) was described in Baral and Rämä (2015). Spores from apothecia growing on decaying *Ascophyllum nodosum* (Class *Phaeophyceae*, Order *Fucales*, Family *Fucaceae*) at the entrance to Portsmouth Harbor, Portsmouth, Hampshire, England, were inoculated and isolated on 0.2SeaMEA (4 g/L malt extract agar with sterile filtered seawater) with antibiotics. The fungus was deposited at the Norwegian marine biobank (Marbank) with the accession number M16FUN0001.

Isolation of *A. encephaloides* TRa018bII (Class *Leotiomycetes*, Order *Helotiales*, Family *Helotiaceae*) was described in Rämä et al. (2014). Spores from a cleistothecium on decaying *Betula* sp*.* (Class *Magnoliopsida*, Order *Fagales*, Family *Betulaceae*) at 70.22874993° N, 19.68153674° E, Troms, Norway, were isolated on 0.2SeaMEA. The fungus was deposited at the Norwegian marine biobank (Marbank) with the accession number M15FUN0043.

### Morphological study

*Emericellopsis* sp. TS7 was incubated on oatmeal agar (OA), potato dextrose agar (PDA) and malt extract agar (MEA) (recipes in Crous et al. (2019)) for 21 days at 25 °C. The cultures where then examined using a dissecting and compound light microscope equipped with differential interference contrast. Morphological characteristics were described and compared to closely related species.

### Growth characterization

Growth requirements of *Emericellopsis* sp. TS7 was characterized by incubation on four different substrates (0.4% malt extract, 0.3% chitin flakes (Sigma), 0.3% fucoidan-rich extracts from *Ascophyllum* and *Fucus* (Non-commercial, Algaia, France) and 0.3% aqueous extract (freeze dried sponge material was macerated and extracted using distilled water for 3 h, the mixture was centrifuged and the aqueous phase was freeze dried. The resulting sample was then fractioned in six fractions and the most polar fraction were used for the agar) from *Stelletta* cf. *normani* (M15034-0-W01, Marbank, Norway), all on 1.5% agar, Sigma) and three different salinities (Distilled water, 50% seawater and seawater) was performed in triplicate. In addition, each medium was incubated at four different temperatures, 2 °C, 10 °C, 15 °C and 25 °C, to determine optimum growth temperature on the different media. The plates were incubated for a total of 43 days. Growths were recorded at day 3, 5, 10, 15, 21, 27, 31, 38 and 43. Distilled water agar (1.5% agar) was used as a control medium.

### Cultivation for nucleic acid extraction

For DNA and RNA extractions, mycelium from liquid seed cultures of *Emericellopsis* sp. TS7, *A. encephaloides* and *C. marina* in 0.2ASME medium (4 g/L malt extract, 40 g/L artificial sea salts (Sigma), MilliQ-water – hereafter MilliQ) were inoculated in 250 mL of the same medium in 1000-mL baffled culture flasks. The media constituents were dissolved in MilliQ. All media were autoclaved at 121 °C for 30 min before inoculation. Incubations were performed at 10–16 °C at 140 rpm (shaking for liquid cultures only). After 13 days the culture was harvested by vacuum filtration through Miracloth (Merck) and the mycelium was subsequently placed in aluminum foil and stored at − 80 °C until processing.

### Isolation of nucleic acids

Genomic DNA from *Emericellopsis* sp. TS7, *A. encephaloides* and *C. marina* mycelium was isolated using Quick-DNA Fungal/bacterial Miniprep Kit (Zymo Research) according to supplier’s instructions. The DNA quality was checked by three methods: First, DNA degradation was checked using gel electrophoresis on 1% TBE (Life technologies) UltraPure agarose (Life technologies) gel stained by GelRed (BioTium) that was run at 180 V for 20 min after loading the samples using Agarose gel loading dye (Amresco). Samples were compared to GeneRuler High Range DNA ladder (ThermoFisher). Secondly, NanoVue Plus (GE healthcare) measurement of wavelength ratio was used to control for contamination and estimate concentration. Finally, Qubit (Invitrogen) measurement using Qubit dsDNA BR Assay Kit (Invitrogen) was used for accurate concentration determination. The DNA sample was stored at − 80 °C.

Total RNA from *Emericellopsis* sp. TS7, *A. encephaloides* and *C. marina* mycelium was isolated using Quick-RNA Fungal/Bacterial Miniprep Kit (Zymo Research) according to the supplier’s protocol. All MilliQ used for RNA extraction were treated with diethyl pyrocarbonate (DEPC - Sigma). Quality control was performed using the same methods as for DNA with the exception of using RiboRuler High Range RNA ladder (ThermoFisher) for gel electrophoresis and Qubit RNA BR Assay Kit (Invitrogen) for concentration determination.

### DNA sequencing and assembly

The draft genomes of *Emericellopsis* sp. TS7, *C. marina* and *A. encephaloides* were sequenced at the DOE Joint Genome Institute (JGI) using Illumina technology. For genome sequencing, 100 ng of DNA was sheared to 300 bp using the Covaris LE220 and size selected using SPRI beads (Beckman Coulter). The fragments were treated with end-repair, A-tailing, and ligation of Illumina compatible adapters (IDT, Inc) using the KAPA-Illumina library creation kit (KAPA biosystems). Illumina Regular Fragment, 300 bp, standard shotgun library (STD) and long insert, 3000 bp, mate pair library (sLMP) were constructed and sequenced using Illumina NovaSeq. All raw Illumina sequence data were filtered for artifact/process contamination using the JGI QC pipeline (Supplementary data 1). An automated attempt was made to reassemble any potential organelle (mitochondrion) from the filtered reads and remove any organelle-matching reads with kmer matching against the resulting contigs with an in-house tool. An assembly of the target genome was generated using the resulting non-Organelle reads with SPAdes v3.12.0 (Bankevich et al. 2012) using the following parameters [−-phred-offset 33 --cov-cutoff auto -t 16 -m 115 –k 25,55,95 --careful]. Similar methodology, employing the UNITE rDNA database (Kõljalg et al. 2013), was used to reassemble the ribosomal DNA from the filtered reads.

Completeness of the euchromatic portion of the genome assemblies were assessed by aligning assembled consensus RNA sequence data with bbtools v38.31 bbmap.sh [k = 13 maxindel = 100,000 customtag ordered nodisk] and bbest.sh [fraction = 85] (Bushnell 2014). This was a routine test by JGI to determine whether significant portions of the genomes were missing.

### RNA library creation, read processing and De novo assembly

For transcriptomics, plate-based RNA sample prep was performed on the PerkinElmer Sciclone NGS robotic liquid handling system using Illumina’s TruSeq Stranded mRNA HT sample prep kit utilizing poly-A selection of mRNA following the protocol outlined by Illumina in their user guide:

https://support.illumina.com/sequencing/sequencing_kits/truseq-stranded-mrna.html, and with the following conditions: total RNA starting material was 1 μg per sample and 8 cycles of PCR was used for library amplification. The prepared libraries were then quantified using KAPA Biosystem’s next-generation sequencing library qPCR kit and run on a Roche LightCycler 480 real-time PCR instrument. The quantified libraries were then multiplexed with other libraries, and the pool of libraries was then prepared for sequencing on the Illumina NovaSeq 6000 sequencing platform using NovaSeq XP v1 reagent kits, S4 flow cell, following a 2 × 150 indexed run recipe.

Raw reads were filtered and trimmed using the JGI QC pipeline resulting in the filtered fastq file (*.filter-RNA.fastq.gz files). Using BBDuk (Bushnell 2014), raw reads were evaluated for artifact sequence by kmer matching (kmer = 25), allowing 1 mismatch and detected artifact was trimmed from the 3′ end of the reads. RNA spike-in reads, PhiX reads and reads containing any Ns were removed. Quality trimming was performed using the phred trimming method set at Q6. Finally, following trimming, reads under the length threshold were removed (minimum length 25 bases or 1/3 of the original read length - whichever is longer).

Filtered fastq files were used as input for de novo assembly of RNA contigs. Reads were assembled into consensus sequences using Trinity (v2.3.2) (Grabherr et al. 2011). Trinity was run with the --normalize_reads (In-silico normalization routine) and --jaccard_clip (Minimizing fusion transcripts derived from gene dense genomes) options.

### Genome annotation and functional annotation

The genome was processed through the JGI Fungal Annotation Pipeline according to the Fungal Genome Annotation Standard Operating Procedure available at https://mycocosm.jgi.doe.gov/programs/fungi/FungalGenomeAnnotationSOP.pdf (Grigoriev et al. 2014). Briefly, gene models were iteratively improved using several gene-predicting tools and comparing it to the RNA transcriptome. Functional annotation was performed using SignalP (Petersen et al. 2011), TMHMM (Krogh et al. 2001), InterProScan (Hunter et al. 2009), SwissProt (Uniprot Consortium 2013) and KOG (Koonin et al. 2004). Finally, KEGG (Kanehisa et al. 2012) hits were used for EC numbers and map to metabolic pathways, while Intepro and SwissProt were used to map gene ontology (GO) terms. Core Eukaryotic Genes Mapping Approach (CEGMA) was used to make a set of reliable genes and determine the completeness of the gene annotation (Parra et al. 2007; Parra et al. 2009).

In addition to the annotations done by JGI, a functional annotation of the Carbohydrate Active Enzymes was performed using the dbCAN2 meta server (Zhang et al. 2018). Annotations were assigned using HMMER (Eddy 2020), Hotpep (Busk et al. 2017) and DIAMOND (Buchfink et al. 2015) on protein FASTA sequence. Domains were assigned by HMMER or if HMMER had no results, by HotPep and DIAMOND as long as both did predicted the same domains. Only genes where two tools had hits were included as recommended by dbCAN2s manual. For comparison with other fungi, two terrestrial and one marine genome were downloaded, *Acremonium chrysogenum* ATCC 11550 (Accession GCA_000769265.1; Terfehr et al. 2014), *Aspergillus niger* (Accession GCA_000230395.2; Andersen et al. 2011) and *Sarocladium strictum* (“*Sarocladium schorii”* Accession GCA_900290465.1*;* Schor et al. 2018). The genome of *S. strictum* only had the assembly available on NCBI, and the annotation was received from the authors (Schor et al. 2018). Furthermore, CAZyme amino acid sequences were extracted and searched against the SulfAtlas database (http://abims.sb-roscoff.fr/sulfatlas/) and the catalytic domain pattern of sulfatases using PROSITE (Sigrist et al. 2002; De Castro et al. 2006; Barbeyron et al. 2016).

The annotated genomes were also uploaded on antiSMASH fungal version (v5.0) to detect biosynthetic gene clusters and assess the biosynthetic potential of the isolates (Blin et al. 2019). Border prediction was manually adjusted; genes with homology to biosynthetic genes or putative tailoring genes were included in the clusters and the clusters were compared to previously published clusters using clinker (Gilchrist and Chooi 2021). Finally, the amount of short simple repeats (SSR) was checked using the Repeat Finder v1.0.1 plugin within Geneious.

### Phylogeny

For *Emericellopsis* sp. TS7: 27 reference sequences including 19 sequences from ex-type strains or cultures were included in the phylogenetic analyses (Supplementary data 2). Sequences for each gene were aligned individually using the E-INS-I and G-INS-I algorithms with PAM100 of MAFFT v7.388 (Katoh et al. 2002; Katoh and Standley 2013) in Geneious Prime v11.0.4 followed by manual adjustment of alignments. The dataset was concatenated in Geneious. PartitionFinder v2.1.1 (Lanfear et al. 2017) was run with the concatenated dataset consisting of the nrDNA genes, 18S, ITS and 28S, and the protein coding genes RNA polymerase II subunit 2 (*rpb2*), transcription elongation factor 1 alpha (*tef1*) and *tub2* with a single intron. For the protein coding regions, each position of the codon was split to different partitions. The PartitionFinder analyses were run with: models MrBayes, linked branchlengths, greedy search, and AICc and BIC model selection criterion (Lanfear et al. 2012). This suggested 12 partitions (using AICc), of varying models (Supplementary data 2). Parallel-MPI MrBayes v3.2.7a with beagle was run for 5.000.000 generations or until average standard deviation of split frequencies was below 0.01 with sampling each 2500 generations with the 12 partitions as suggested by ModelFinder (Ronquist et al. 2012). In addition, PhyML 3.0 was run from the webserver as a single partition with smart model selection using AIC, SPR tree search improvement and aBayes and aLRT SH-like fast likelihood-based branch support search (Anisimova and Gascuel 2006; Guindon et al. 2010; Anisimova et al. 2011; Lefort et al. 2017). The model selected was GTR + I + G. The maximum-likelihood tree using aBayes can be found in Supplementary data 3.

For *C. marina* and *A. encephaloides*, the 15 gene datasets from Johnston et al. (2019) containing 265 taxa were downloaded and the genes from *C. marina* and *A. encephaloides* were aligned to each individual gene alignment before it was concatenated to a single multilocus dataset. The dataset from Johnston et al. (2019) was modified slightly by removing a few introns from protein coding genes and cutting edge alignments only present in a minority of sequences. The alignment was loaded into IQ-TREE v1.6.12, each gene with its own partition (Nguyen et al. 2014). IQ-TREE was run with the parameters [−m MFP –bb 10,000 –alrt 10,000 –nt AUTO], such that it selected the best model for each partition using ModelFinder (Kalyaanamoorthy et al. 2017), performed 10,000 ultrafast bootstraps (Minh et al. 2013) and 10,000 SH-aLRT branchtests (Guindon et al. 2010).

## RESULTS

### Genome features of *Emericellopsis* sp. TS7

The *Emericellopsis* sp. TS7 genome was assembled into 114 scaffolds, with a total size of 27.3 Mbp, Table 1. Mapping of RNA-Seq reads and de novo assembled contigs revealed that 99.2 and 97.3%, respectively, mapped back to the genome. The mitochondrial genome was separately assembled into a single scaffold of 25,688 bp and is likely to be circular. The genome characteristics of *A. encephaloides* and *C. marina* are presented at the end of the results section.

**Table 1:** Overview of genome assembly and gene statistics for *Emericellopsis* sp. TS7, *Calycina marina* and *Amylocarpus encephaloides*

Isolates	*Emericellopsis* sp. TS7	*C. marina*	*A. encephaloides*
Genome statistics			
Genome assembly size (Mbp)	27.3	34.21	46.29
Coverage	225.6	185.26	127.83
# of scaffolds	114	1318	2381
# of scaffolds > = 2 k	105	1168	1600
Scaffold L50	14	173	168
Scaffold N50 (Mbp)	0.76	0.05	0.07
# of gaps	22	37	68
% of scaffold length in gaps	0.0	0.0	0.0
Largest scaffold	1.47	0.38	0.42
% GC	54.2	47.6	44.9
Transcriptome and gene models			
EST mapped to genome (%)	99.2	98.8	99.0
Average gene length	1832	1758	1770
exons per gene	2.59	3.13	3
# of gene models	9964	9558	11,869
Genes/Mbp	364.98	279.39	256.41
CEGMA (%)	99.34	99.34	99.54
BGCs	35	21	34
CAZyme genes	396	217	356
KOG annotated	5201	4723	5413
KEGG annotated	1969	1670	2041

### Gene features and functional annotation of *Emericellopsis* sp. TS7

The 9964 predicted gene models gave a gene density of 365 genes/Mbp. CEGMA estimated that 99.34% of the core genes were present, which indicates a nearly complete genome. There were 162 tRNAs and a single complete nrDNA region in the assembly. A total of 4331 (43%) genes were generically annotated with hypothetical (3252) or expressed (1079) proteins. The MAT-1-1 mating locus associated with sexual reproduction was also identified via BLAST in the assembly.

A total of 5201 (52%) genes were recognized as orthologous genes based on hits in the KOG database (Table 1), of these 1317 (25%) received general functional predictions or were conserved genes with unknown functions (Supplementary data 4). This indicates that 4763 of the 9964 (47.8%) predicted genes do not have characterized orthologs or are lineage specific genes. A small portion of these genes may be pseudogenes that are not functional or genes that have been incorrectly predicted from the annotation pipeline. The largest group of identified orthologs belonged to the posttranslational modification, protein turnover and chaperones category (483). Signal transduction (377), energy production and conversions (323), carbohydrate transport and metabolism (318) and translation, ribosomal structures and biogenesis (317) were the next four highly represented categories. Secondary metabolite biosynthesis, transport and catabolism (268) made up 2.5% of the functionally annotated orthologs.

Of the 9964 genes, only 1969 were classified based on the KEGG database, Table 1. The largest group of these were enzymes with known functions but undetermined pathways (688) (Supplementary data 4). This was followed by enzymes involved in amino acid metabolism (618), carbohydrate metabolism (433), metabolism of complex carbohydrates (314), and biodegradation of xenobiotics (298). Pathways associated with biosynthesis of secondary metabolites had 99 enzymes assigned to it.

### Phylogenetic placement of *Emericellopsis* sp. TS7

**Fig. 1:**
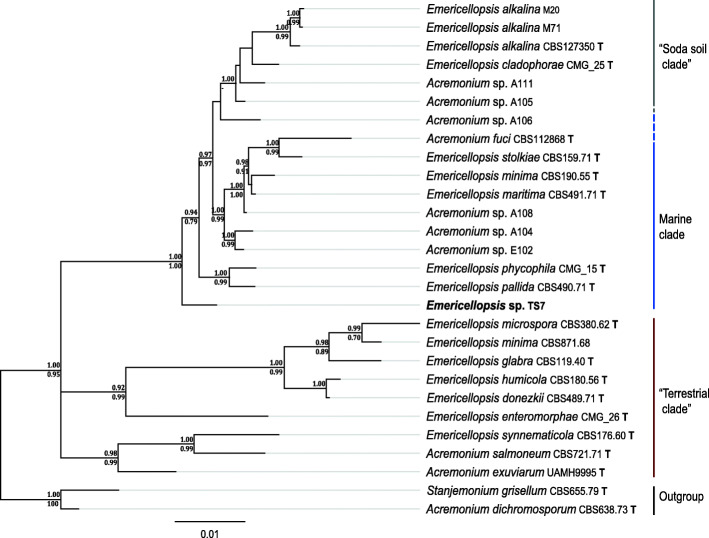
Phylogenetic tree from MrBayes of the genus *Emericellopsis* based on a six gene multilocus alignment of available ex-type and representative sequences. Branch support values are from Bayesian posterior probability (top) and Maximum-likelihood aBayes support test (bottom). Branch length represents substitutions per sequence site. The taxon in bold is the studied fungus. The bold letter T denotes sequences of ex-type cultures. Accession numbers for each isolate are in Supplementary data 2, PhyML tree can be seen in Supplementary data 3

Preliminary ITS analysis and morphological characterization indicated that *Emericellopsis* sp. TS7 was likely a novel species and for this reason, a thorough multigene phylogenetic analysis was performed. A concatenation of nuclear nrDNA 18S, ITS and 28S, and the protein coding genes *rpb*2, *tef1* and *tub*2 were made and run through MrBayes using 12 partitions with different models as suggested by PartitionFinder and PhyML using the smart model selection (Supplementary data 2). The *Acremonium/Emericellopsis* species split into three clades; terrestrial soil, marine, and alkaline or “soda soil” (Fig. 1) as previously reported by Grum-Grzhimaylo et al. (2013). *Emericellopsis* sp. TS7 was grouped in the marine clade as an early branch, closest to *E. pallida and E. phycophila* with maximum support values. All three major ecological clades have support in both Bayesian and maximum-likelihood models, while individual taxa and branches in some cases have different branching in Bayesian and maximum-likelihood trees. The terrestrial clade have long branches and polytomy, but it is also the clade with the largest portion of missing data (70.1% - missing 18S, *rpb2* and *tef1*) compared to the marine and alkaline clade (20.1% missing data). The alkaline clade contains *E. cladophorae* that was isolated from marine algae. *Emericellopsis donezkii* and *E. enteromorphae* were isolated from fresh water and marine algae, respectively. The three species, *E. cladophorae*, *E. donezkii* and *E. enteromorphae*, were all isolated from marine sources, but they do not group in the marine clade. However, all three lack sequence information for 18S, 28S, *rpb2* and *tef1*.

### Growth characterization of *Emericellopsis* sp. TS7

**Fig. 2:**
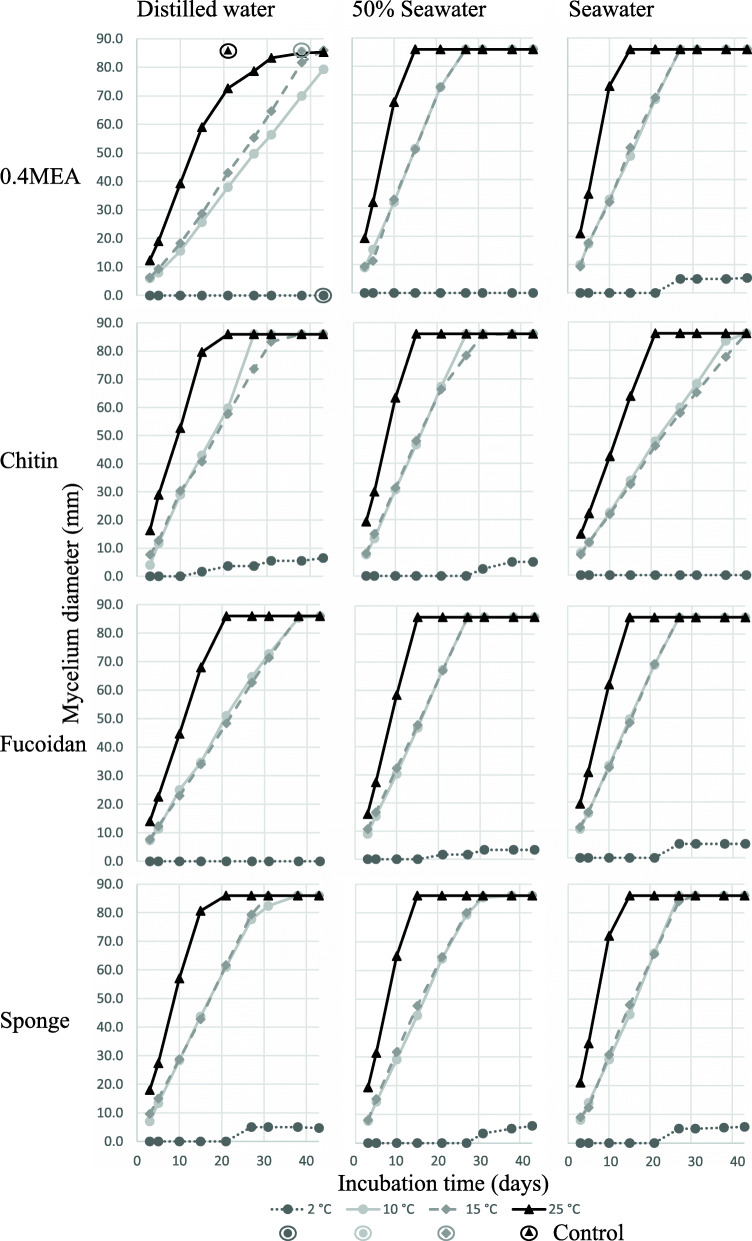
Growth characterization of *Emericellopsis* sp. TS7 using four different substrates and three different salinities incubated at four different temperatures. Maximum growth was 86 mm. Max growth of growth control on distilled water agar is shown in the first panel with encircled symbols. The control for 10 °C and 15 °C is identical

In order to examine the growth characteristics of *Emericellopsis* sp. TS7, the isolate was grown on different substrates, salinities and temperatures (Fig. 2). The fastest growth rate occurred at 25 °C for all substrates and salinities. The preferred substrate was MEA and sponge extract, prepared with seawater. The slowest growth occurred on MEA prepared with distilled water. Generally, growth on media prepared with distilled water was slower compared to salt containing media. Growth at 2 °C occurred for all salinities with sponge extract. *Emericellopsis* sp. TS7 on the control medium reached full growth within 21 days at 25 °C, 38 days at 10 °C and 15 °C, and no growth at 2 °C. Growth on 0.4MEA medium without salt and chitin medium with salt was slower than the control medium.

### CAZymes and other industrially relevant genes

**Fig. 3:**
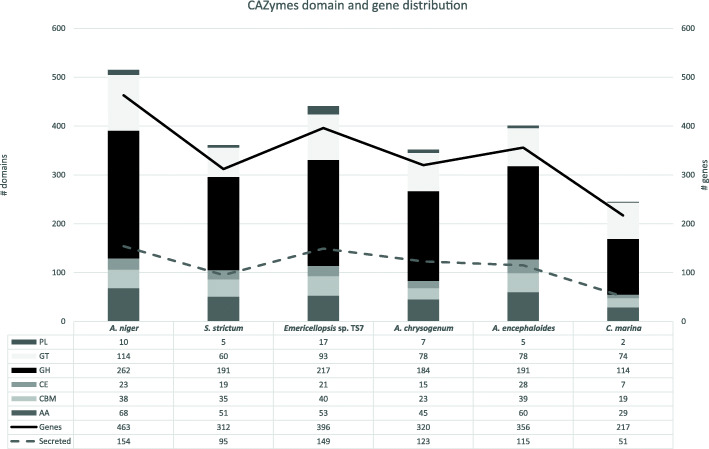
Overview of the distribution of CAZymes in *Emericellopsis* sp. TS7 *Amylocarpus encephaloides* and *Calycina marina* and three other fungi. The lines indicate the number of genes and number of genes with putative secretion signal and uses the secondary Y-axis

**Fig. 4:**
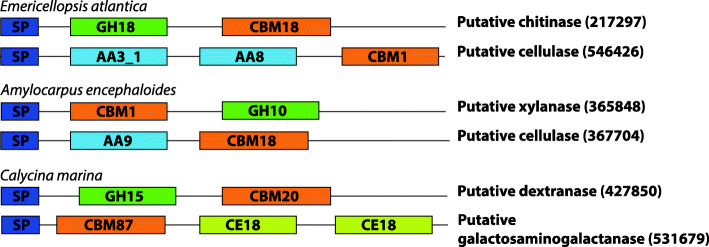
Examples of putatively secreted modular CAZymes from *Emericellopsis* sp. TS7, *Amylocarpus encephaloides* and *Calycina marina*. The illustration is not to scale. SP – Secretion signal peptide, GH – Glycoside hydrolase, CBM – Carbohydrate binding module, AA – Auxiliary activity, CE – Carboxyl esterase. Number indicates enzyme class. Number in brackets is protein identifier

The number of CAZymes in *Emericellopsis* sp. TS7 was 396 (3.97% of total genes), of which 149 possessed secretory peptide signal indicating that they are likely to be secreted into the external environment or across other membranes (38% of CAZymes). A comparison of *Emericellopsis* sp. TS7, *A. encephaloides* and *C. marina* with three other fungal genomes, namely *A. niger*, *S. strictum* and *A. chrysogenum* (two terrestrial/pathogens and one from sewage water outlet to the sea) indicated that *Emericellopsis* sp. TS7 had the second highest number of CAZyme genes (Fig. 3). A relatively high number of CAZymes in *Emericellopsis* sp. TS7 and *A. chrysogenum* had a secretory signal compared to the other species (38% vs 23–33%). *Emericellopsis* sp. TS7 had a higher number of polysaccharide lyase (PL), glycosyl transferase (GT) and GH domains compared to the other marine isolates. *Amylocarpus encephaloides* on the other hand contained the highest number of carboxyl esterases (CEs), carbohydrate binding modules (CBMs) and auxiliary activity (AA) domains of the marine fungi. *Calycina marina* contained two PL8 (absent in the other studied fungi), which act on uronic acid, a common constituent of seaweeds (Ponce et al. 2003; Sánchez-Machado et al. 2004). CAZyme genes are often modular with many genes containing one or more enzymatic domains along with CBMs that bind to substrates and have no catalytic function. Examples of this are the putatively secreted CAZyme gene 217,297 in *Emericellopsis* sp. TS7 with a GH18 and CBM18 domain (putative chitinase) or 546,426 (putative cellulase) with a CBM1, AA3_1 and AA8 domain (Fig. 4).

The different classes of CAZymes followed a similar putative secretion signal pattern in the fungi compared here (Supplementary data 5). Generally, few genes (4–6%) with predicted GT activity contained putative signal peptide for secretion, but these are often involved in intracellular synthesis. Genes with PL activity contained secretion signal in 80–88% of cases, with the exception in *C. marina* and *S. strictum* that only had signal in 50 and 60% of genes, respectively. *Amylocarpus encephaloides* had the highest ratio of CBM containing genes with secretion signal (66.7%) and *C. marina* had the lowest ratio of genes with secretion signal for all classes except GHs. For example, *Emericellopsis* sp. TS7 genes with AA had secretion signal in 42.3% of cases, CBM in 55.0%, CE in 66.7%, GH in 42.5%, GT in 6.7% and PL in 88.2%.

The domains that occurred in the highest numbers across the six genomes analyzed were associated with cellulose, hemicellulose, xylan, mannose, fucose, pectate, and chitin. In the secreted enzymes mainly cellulose-, chitin- and xylan-interacting domains were abundant. The unclassified domain GH0 was found in *Emericellopsis* sp. TS7 (1), *C. marina* (1) and *A. encephaloides* (2). In total, *Emericellopsis* sp. TS7 had 176 different classes of CAZymes (Supplementary data 5).

*Emericellopsis* sp. TS7 does not appear to possess genes encoding polyphenol oxidases or fucoidanase, but does have genes encoding fucosidase (GH29 and GH95), a fucose transporter and a few GTs with potential fucose activity (GT1 and GT31). In addition, *Emericellopsis* sp. TS7 also contains seven potential sulfatase genes based on the sulfatase catalytic site pattern (Barbeyron et al. 2016), but none of the domains are on CAZymes.

The gene for the industrially relevant enzyme phytase was also found (Lei et al. 2013) in *Emericellopsis* sp. TS7, *C. marina* and *A. encephaloides*, along with histidine acid phosphatases that share the same enzyme classification (EC 3.1.3.8) with phytase.

### Biosynthetic gene clusters of *Emericellopsis* sp. TS7

**Fig. 5:**
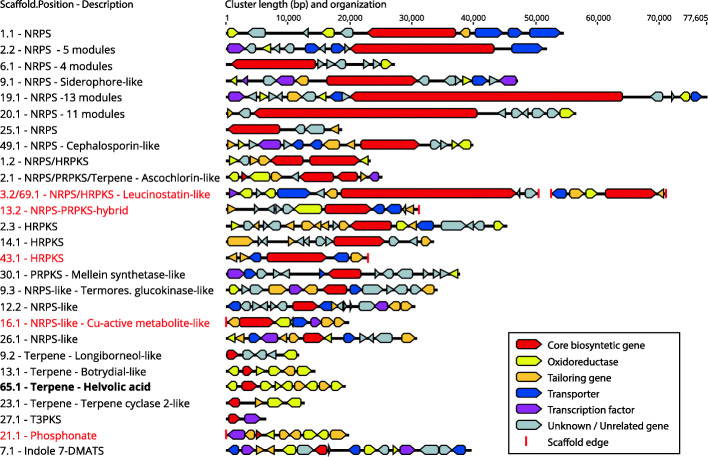
Overview of BGC structure of the predicted clusters in *Emericellopsis* sp. TS7 colored after function. Clusters marked in red were on the end of scaffolds and may be incomplete. The leucinostatin-like cluster was split in two, but is presented as one cluster with a gap. Helvolic acid, produced by the cluster in bold, were detected in MS analyses of fermentation broths

A total of 35 biosynthetic gene clusters (BGCs) were predicted using antiSMASH, with 27 of these gene clusters being shown in Fig. 5. Eight are not included in the figure because they were solitary core genes not surrounded by other tailoring, transport or transcription genes or they were likely precursor genes in sterol synthesis such as the squalene and lanosterol synthase. The clusters contained a range of oxidoreductases, transcription factors, tailoring genes and transporters together with core biosynthetic gene(s). These BGCs included eight NRPS clusters, six NRPS-like clusters, nine terpene clusters, six polyketide synthase (PKS) clusters, three mixed NRPS-PKS clusters, one hybrid NRPS-PKS cluster, one phosphonate cluster and one indole cluster.

Several of the clusters had homology to known clusters according to KnownClusterBlast, these were further investigated by a synteny analysis using clinker (Gilchrist and Chooi 2021). Only the BGC for ascochlorin (Araki et al. 2019), leucinostatin A/B (Wang et al. 2016), botrydial (Pinedo et al. 2008), cephalosporin C (Terfehr et al. 2014) and helvolic acid (Mitsuguchi et al. 2009) showed a high degree of conserved genes in the *Emericellopsis* clusters (Supplementary data 6). Several of the NRPS-genes without homologous hits had a configuration of 4–13 modules according to antiSMASH.

*Emericellopsis* sp. TS7 was cultivated in several different media and the fermentation broths were extracted. The resulting fractions from the extracts showed antibacterial activity against *Enterococcus faecalis*, *Streptococcus agalactiae* and *Staphylococcus epidermidis*. No toxicity was detected against A2058 human melanoma cancer cells. Methods and details of bioactivity experiments can be found in Supplementary data 7.

### Genome description of *Calycina marina*

The genome assembly of *C. marina* was more fragmented when compared to *Emericellopsis* sp. TS7. The assembly statistics reveal that the L50 was 173 with an N50 of 50 kbp and the final assembly consisted of 1318 scaffolds with a total length of 34.2 Mbp. The number of predicted genes in *C. marina* was 9558, which was slightly fewer than in *Emericellopsis* sp. TS7 despite the fact that *C. marina* has a larger genome. *Calycina marina* distinguished itself from the other genomes analyzed in having comparatively few CAZyme genes, totaling 217; and the lowest proportion of potentially secreted CAZyme genes at 51 (24% of CAZymes). The genome contained 21 potential BGCs distributed as nine NRPS/NRPS-like, five PKS (including two type 3), three terpene, one indole, one hybrid, one aromatic prenyltransferases and one ribosomally synthesized and post-translationally modified peptide (RiPP).

### Genome description of *Amylocarpus encephaloides*

The genome assembly of *A. encephaloides* was also more fragmented than *Emericellopsis* sp. TS7 with an L50 value of 168 and N50 of 74 kbp. The genome assembly consisted of 2381 scaffolds with a total length of 46.3 Mbp, which was larger than that for *Emericellopsis* sp. TS7 and *C. marina.* The total number of predicted genes was 11,869, which is the highest number among the three sequenced strains. Despite being fragmented, the genome was complete in terms of core gene presence with a CEGMA value of 99.56%. *Amylocarpus encephaloides* had 356 CAZyme genes, of which 115 are potentially secreted. The genome showed a higher portion of CAZyme genes with CBM1 (Cellulose binding) modules and secretion of these (15 genes, 80% secreted). *Amylocarpus encephaloides* also had the largest portion of CBM containing CAZymes with secretion signal in total (66.7%). A total of 34 BGCs were detected in the genome, distributed as 14 PKS (one type 3), 10 NRPS/NRPS-like, five terpene, four hybrid clusters and one RiPP.

### Phylogenetic placement of *Amylocarpus encephaloides* and Calycina marina within Helotiales

**Fig. 6:**
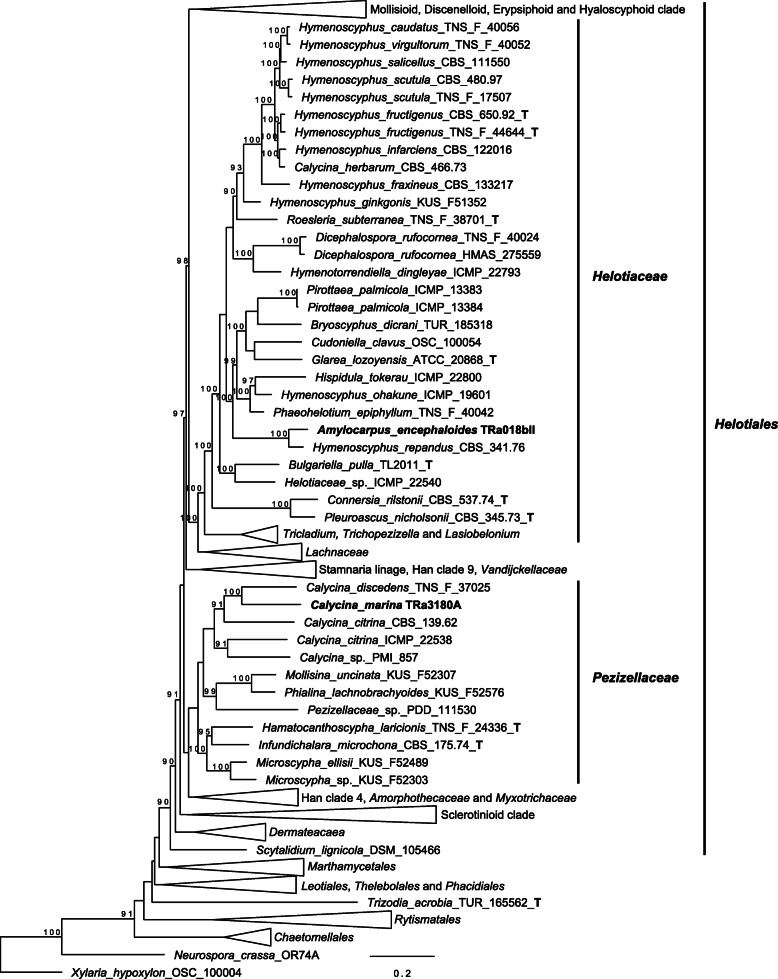
Phylogeny of *Helotiales* based on a 15-gene dataset for the analysis. The support values are from the ultrafast bootstrap in IQ-TREE. The bold letter T denotes ex-type sequences. *Xylaria hypoxylon* was used as an outgroup

A 15-gene multilocus phylogenetic analysis was performed using a slightly modified dataset of Johnston et al. (2019). *Calycina marina* was placed together with the rest of *Calycina* within *Pezizellaceae*, where it formed a monophyletic clade (Fig. 6). *Amylocarpus encephaloides* was placed within *Helotiaceae* on a branch with *“Hymenoscyphus” repandus*. “*Hymenoscyphus” repandus* was not placed together with the rest of the *Hymenoscyphus* that formed a distinct monophyletic clade. Both of these clades were within *Helotiales*, sensu Johnston et al. (2019).

## TAXONOMY

***Emericellopsis atlantica*** L.W. Hou, Crous, Rämä & Hagestad, **sp. nov.**

MycoBank MB838493

Fig. 7

**Fig. 7:**
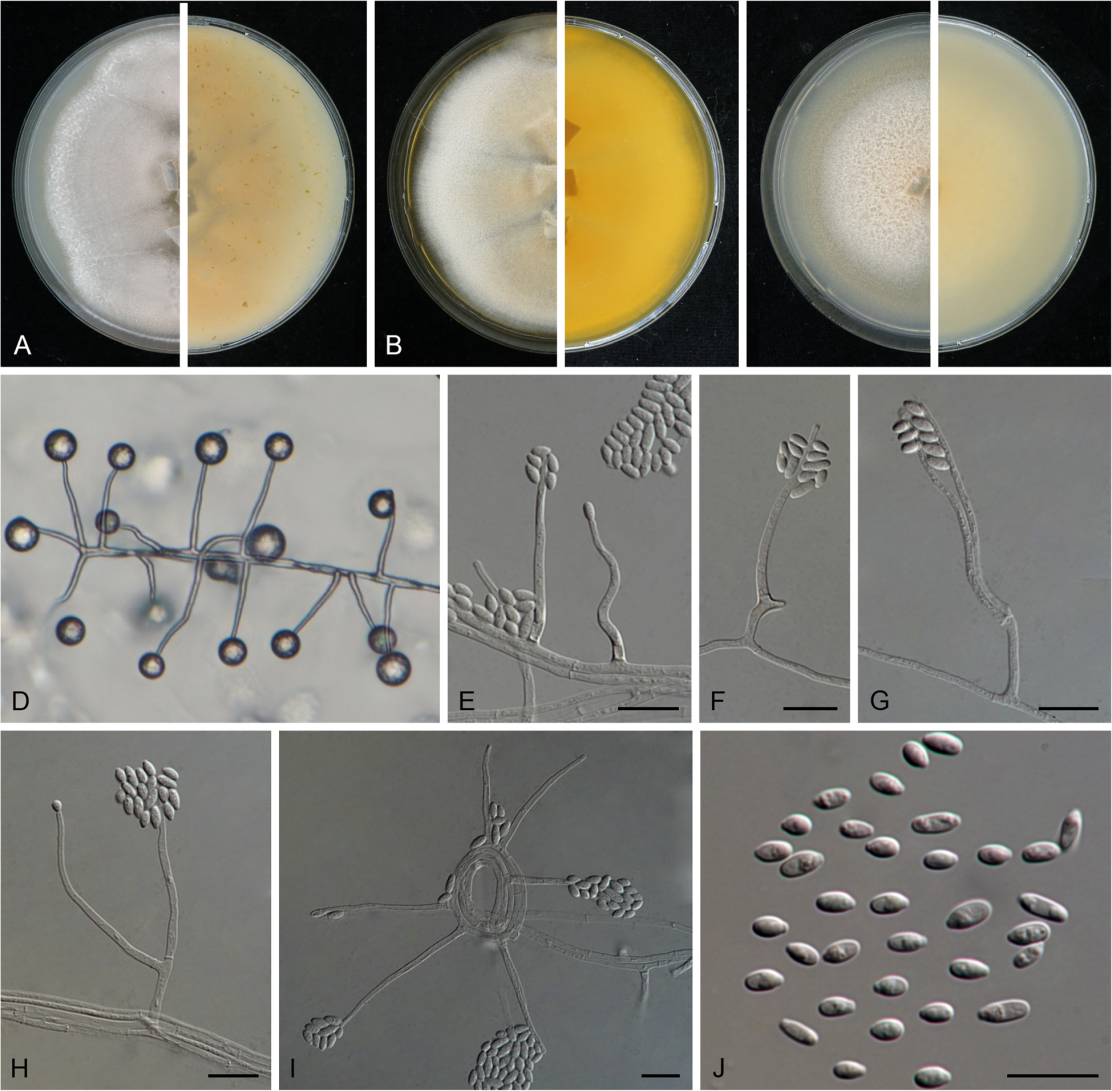
*Emericellopsis atlantica* (ex-type CBS 147198). Colonies on OA (**a**), MEA (**b**) and PDA (**c**) after 21 d at 25 C. **d–f, i** Phialides. **g–h**. Branched conidiophores. **j** Conidia. Scale bars = 10 μm

***Etymology*:** *atlantica*, referring to the Atlantic Ocean where the fungus was found.

***Diagnosis:*** *Emericellopsis atlantica* can be distinguished by the production of conidia with irregular-shaped guttules, and longer phialides measuring 24.5–50(− 64) μm. Furthermore, *E. atlantica* occasionally produces branched conidiophores. *Emericellopsis atlantica* differed by its longer conidiogenous cells, which were 19.0 ± 7.5 × 1.5 ± 0.5 μm in *E. enteromorphae*. Colonies of *E. atlantica* also grew faster than the three other marine species (Gonçalves et al. 2020).

***Typus***: Ireland, from 1350 m depth in the Atlantic Ocean (54.0613 N, 12.5518 W), from the sponge *Stelletta normani*, 16 June 2010, T.D.S. Sutton (holotype CBS H-24579, ex-type living culture TS7 = CBS 147198).

***Description***: Colonies after 21 d incubation at 25 °C: On OA reaching 65 mm diam., flat, entire margin, dusty and rosy buff at centre, dirty white at periphery, reverse ochreous. On MEA reaching 70 mm diam., flat, entire margin, felty, pale ochreous at centre, dirty at periphery, reverse ochreous. On PDA reaching 80 mm diam., flat, entire margin, cottony, rosy buff at centre, buff at periphery, reverse buff. *Mycelium* consisting of branched, septate, hyaline, smooth- and thin-walled hyphae, up to 2 μm wide. *Conidiophores* arising from submerged or superficial hyphae, sometimes radiating out from sterile coils formed by the mycelium, (sub-)erect or slightly curved, simple or poorly branched, ca. up to 66 μm long, 1.5–3 μm wide at the base, hyaline, smooth-walled, with cell walls usually thicker than those of the vegetative hyphae. *Conidiogenous cells* integrated, monophialidic, terminal, lateral, straight to slightly flexuose, cylindrical, 24.5–50(− 64) μm long, 1.5–2.5 μm wide at the base, with inconspicuous collarette and periclinal thickening at the conidiogenous locus, hyaline, thick- and smooth-walled branched conidiophores. *Conidia* formed in globose slimy heads at the apex of phialides, obovoid or ellipsoidal with truncate base, aseptate, hyaline, thin- and smooth-walled, 3–6(− 9) × 2–2.5 μm, with 1–2 irregular shaped guttules. *Chlamydospores* not observed.

*Sexual morph* not observed.

***Habitat/host:*** Only known from type. Isolated from the sponge *Stelletta normani*, collected in a marine environment.

***Distribution:*** Currently unknown.

***Notes:*** *Emericellopsis atlantica* is represented by a single isolate that clusters on a solitary branch basal to the clade containing the “Marine clade” and “Soda soil clade” of *Emericellopsis*. Morphologically, *E. atlantica* can be distinguished from other *Emericellopsis* spp. with comparable conidiophores and conidia by producing longer conidiogenous cells and conidia with irregular-shaped guttules. Currently there are 26 entries listed in the genus *Emericellopsis* in Index Fungorum, including four varieties, *E. terricola* var. *glabra*, *E. terricola* var. *terricola, E. synnematicola* var. *magna* and *E. synnematicola* var. *synnematicola* (Van Beyma Thoe Kingma 1939; Mathur and Thirumalachar 1960; Backus and Orpurt 1961). Morphologically, *Emericellopsis atlantica* is distinct from other species in having longer conidiogenous cells, 24.5–50(− 64) μm, which tend to be shorter than 50 μm in most species, except for *E. donezkii*, *E. koreana*, *E. microspora*, *E. mirabilis*, *E. pusilla* and *E. robusta,* which again lack conidia with irregular-shaped guttules. Furthermore, conidia of *E. atlantica* (3–9 × 2–2.5 μm) are longer than those of *E. donezkii* (2.5–6.5 × 2–2.5 μm) and *E. koreana* (3–5 × 1.5–2.5 μm), and smaller than *E. microspora* (3.0–10.0 × 1.5–4.0 μm), *E. mirabilis* (6–11 × 2.5–3 μm), *E. pusilla* (4.5–10.5 × 2.25–4.5 um) and *E. robusta* (13–17.5 × 3.8–4.5 um) (Malan 1952; Stolk 1955; Backus and Orpurt 1961; Mathur and Thirumalachar 1962; Gams 1971; Belyakova 1974; Phookamsak et al. 2019). For comparisons with morphologically similar and phylogenetically related species, see Table 2.

**Table 2:** Morphological comparison of *Emericellopsis* spp.

Species	Colonies	Growth rate (mm/d)	Conidiogenous cells/Conidiophores	Conidial shape and size	References
*E. atlantica* sp. nov.	On PDA reaching 74 mm at 14 d; on OA reaching 56 mm at 14 d; on MEA reaching 58 mm at 14 d,	4.0–5.3	24.5–50(− 64) × 1.5–2.5 μm	obovoid or ellipsoidal with truncate base, 3–6(− 9) × 2–2.5 μm, with 1–2 irregular shaped guttules	This study
*E. alkalina*	On MEA (PH = 6.5) growing slower, reaching 32–38 mm diam in 10 d.	3.2–3.8	20–35 × 1.5–1.8 μm, sometimes lateral branches form	narrowly ellipsoidal, 3.5–6 × 1.8–2.2 μm	Grum-Grzhimaylo et al. (2013)
*E. cladophorae*	On OA reaching 36 mm in 21 d	1.7	21.0 ± 5.0 × 2.0 ± 0.5 μm, sometimes lateral branches form	circular to oblong-ellipsoidal, 5.5 ± 2.5 × 3.5 ± 1.5 μm	Gonçalves et al. (2020)
*E. donezkii*	On PDA reaching 30 mm in 9 d	3.3	(20–) 30–45 (−80) × 1.5–2 μm	ovoid-cylindical, ellipsoidal or sometimes inaequilateral subincurved, (2.5–) 4–5.2 (− 6.5) × 2–2.5 μm	Belyakova (1974)
*E. enteromorphae*	On OA reaching 42 mm in 21 d	2	19.0 ± 7.5 × 1.5 ± 0.5 μm, mostly simple orthotropic	cylindrical to narrowly ellipsoidal, 4.5 ± 1.0 × 2.0 ± 0.5 μm	Gonçalves et al. (2020)
*E. humicola*	–	–	20–45 × 1.5–2.5 μm, producing a succession of phiaIospores	ellipsoidal, 5–8 (− 10) × 2.0–3.5 μm	Cain (1956); Grosklags and Swift (1957)
*E. koreana*	On PDA reaching 15.5 mm in 7 d; on OA reaching 17 mm in 7 d; on MEA reaching 27.5 mm in 7 d	2.2–3.9	(15.5–)31.5–40(− 59) × 2(− 2.5) μm	ellipsoidal or oblong-ellipsoidal, 3–4(− 5) × 1.5–2(− 2.5) μm	Phookamsak et al. (2019)
*E. maritima*	On OA and MEA reaching 35–40 mm after 9–10 d.	3.5–4.5	(17–)20–26(−29) × 1–2 μm	ellipsoidal-piriform, 6.5–8.0(− 9.0) × (2.0–)2.5–3.3(− 4.0) μm	Belyakova (1970)
*E. microspora*	On Czapek Dox agar, reaching 40 mm after 14 d	2.9	25–60 × 2–3 μm (up to 95 μm long), mostly unbranched, usually septate near the base	ovoid to ellipsoidal, 3.0–10.0 × 1.5–4.0 μm	Backus and Orpurt (1961)
*E. minima*	On OA reaching 30 mm in 7 d	4.3	20–30 × 2–2.5 μm	ellipsoidal, 4–10 × 2–3.5 μm	Stolk (1955)
*E. mirabilis*	–	–	30–50 × 3 μm	oblong-ellipsoidal, or of cylindrical one-guttulate, 6–11 × 2.5–3 μm, usually 8 × 3 μm	Malan (1952); Stolk 1955
*E. pallida*	On OA reaching 35 mm in 9 d	3.9	25–45 × 1.5–2 μm	ovoid to ovoid-cylindrical, (3.2–) 4–5.2 (− 7.8) × 1.5–2.5 (− 3.3) μm	Belyakova (1974)
*E. persica*	On MEA growing slower, reaching 17–18 mm diam in 7 d	1.9–2.0	30–40 × 2–2.5 μm	narrowly ellipsoidal, 4.5–6.5 × 2 μm, adhering in slimy heads	Hyde et al. (2016)
*E. phycophila*	On OA reaching 36 mm in diam in 21 d	1.7	21.0 ± 5.0 × 2.0 ± 0.5 μm, lateral branches form	circular to oblong-ellipsoidal, mean ± SD = 5.5 ± 2.5 × 3.5 ± 1.5 μm	Gonçalves et al. (2020)
*E. pusilla*	–	–	33–79 × 1.5–2.5 μm, conidiophores developing as lateral branches on subaerial hyphae, long, erect, slender, spirally produced	oblong, ovoid to obpyriform, 4.5–10.5 × 2.25–4.5 μm	Mathur and Thirumalachar (1962)
*E. robusta*	Colonies 18–20 mm diam in 10 d	1.8–2.0	20–65 × 2.5–5.0 μm, phialides simple or with whorled branching, with a short apical wall thickening	cylindrical, slightly truncated at the base, 13–17.5 × 3.8–4.5 μm, L / W 3.5–3.9	Gams (1971)
*E. salmosynnemata*	–	–	14.0–38 × 0.75–1.5 μm, conidiophores arising along entire length of synnemata, septate only at base, unbranched	ellipsoidal or ovoid, 3.4–6.4 × 2.3–3.4 μm	Grosklags and Swift (1957)
*E. sphaerospora*	On OA agar reaching 55–60 mm in 2 wk	3.9–4.3	16–32 × 2–3 um, simple, discrete, awl-shaped, with no distinct coliarette	ellipsoidal to cylindrical or allantoid, 3–8 × 1.5–3 um, aggregating in slimy heads	Udagawa and Furuya (1988)
*E. stolkiae*	On CMA agar reaching 40–44 mm in 14 d	2.9–3.1	18–40 × 3–3 μm, mostly unbranched, rarely forked	ovoid, 3.5–8.4 × 2.2–5 μm, most often 4.3–5.7 × 3.3–3.8 μm	Davidson and Christensen (1971)
*E. synnematicola*	–	–	conidiophores arising from synnemata, 2–3 mm long	ovoid-ellipsoidal, 3.5–6.5 × 2–3 μm	Mathur and Thirumalachar (1960)
*E. synnematicola* var*. mangus*	Reaching 40 mm in 15 d	2.7	synnemata erect, distributed uniformly in the colony, up to 4 mm high, cream-coloured, bearing prominent slimy conidial heads; conidiophores simple or branched	globose to subglobose, 2.5–7.5 μm	Leelavathy (1966)
*E. terricola*	On OA reaching 5–6 cm after 2 mo	0.8–1.0	30–40 × 2–3 μm	ellipsoidal or ovoid, one or two oil droplets, 6–8 × 3.3–4 μm	Van Beyma Thoe Kingma (1939)

*Emericellopsis atlantica* is also morphologically similar to some species belonging to *Acremonium* sect. *Simplex* (Gams 1971). However, these species have been transferred to *Sarocladium* (*S*. *bactrocephalum*, *S. glaucum, S*. *kiliense*, *S. ochraceum*, *S. strictum;* Summerbell et al. 2011), *Parasarocladium* (*P. breve*; Summerbell et al. 2018), or were clearly phylogenetically distant from *Emericellopsis* (Summerbell et al. 2011) based on phylogenetic analysis using nrDNA and actin sequences. Only *A. larvarum* lacks cultures or DNA sequence data but can be distinguished in having shorter conidiogenous cells (14–22 μm), and producing synnemata (Gams 1971). Other “*Acremonium*” species that clustered in *Emericellopsis* include *A. fuci*, *A. salmoneum* and *A. exuviarum*, but they proved to be phylogenetically distant from *E. atlantica* (Gams 1975; Sigler et al. 2004; Zuccaro et al. 2004; Summerbell et al. 2011).

***Material examined:*** Ireland, from 1350 m depth in the Atlantic Ocean (54.0613 N, 12.5518 W), from the sponge *Stelletta normani*, 16 June 2010, T.D.S. Sutton (holotype CBS H-24579, ex-type living culture TS7 = CBS 147198).

## DISCUSSION

### The driftwood-associated *Amylocarpus encephaloides* and seaweed-associated *Calycina marina*

*Amylocarpus encephaloides* and *C. marina* are both considered obligate marine fungi and are therefore of significant interest in sequencing campaigns. *Amylocarpus encephaloides* had the largest genome of the three genomes presented here, with about 2000 more genes than the two other fungal genomes. Despite the high gene count, *A. encephaloides* had fewer CAZymes than *E. atlantica*, but a higher the number of secreted CAZymes containing CBM1 and CBM87 compared to the other genomes. CBMs are important for binding to insoluble substrates such as cellulose (Boraston et al. 2004; Zhao et al. 2014). In addition, *A. encephaloides* had a higher number of CEs and AAs than the two other genomes. Specifically, it had seven AA1 laccases acting on phenolic substrates and can be involved in lignin degradation. AAs are often associated with degradation of lignin and the high amounts of CBMs, AAs and CEs shows an adaptation towards woody substrates that sporocarps of *A. encephaloides* are exclusively found on.

*Calycina marina* had the lowest number of genes, despite having a larger genome than *E. atlantica*. *Calycina marina* also had the fewest BGCs and a significantly smaller amount of CAZymes relative to the total gene count. The fungus had two CAZymes that were members of PL8 family. This class is acting on uronic acid which is a constituent of some types of fucoidan (Ponce et al. 2003). This indicates an adaptation towards living from the preferred substrate of occurrence. The fungus *C. marina* is found on decaying macroalgae in the upper part of the tidal zone. The quick colonization suggests that this fungus is already present in or on the algae before it is washed ashore (Baral and Rämä 2015). The relation of *C. marina* to its algal host needs to be examined more closely to determine of it is found inside (endophyte) or on the surface (epiphyte) of the algae before it is washed ashore using for example metagenomics studies.

The genome scaffolds of *A. encephaloides* and *C. marina* were significantly more fragmented than the assembly of *E. atlantica*. This is potentially due to a larger portion of repetitive elements which can complicate genome assemblies (Sotero-Caio et al. 2017; Tørresen et al. 2019). CEGMA analysis indicated that nearly all core genes were accounted for, so the genome can be considered complete with respect to core gene content. However, the many small contigs made it impossible to identify complete BGCs since many were located at the contig edges. The fragmentation could also reduce the number of BGCs that were detected.

### The genome of Emericellopsis atlantica

The size of the genome assembly of *Emericellopsis atlantica* was 27.3 Mbp. The assembly was approximately 3 Mbp smaller than the average genome size of karyotyped *Acremonium* (Walz and Kück 1991) and 1.3 Mbp smaller than the sequenced *A. chrysogenum* ATCC 11550 (Terfehr et al. 2014). However, these *Acremonium* species, *A. chrysogenum*, *S. strictum*, *A flavum* and *Cephalosporium polyvaleurum*, are not part of the *Emericellopsis* clade.

The G + C content of *E. atlantica* was 54.2%, which is higher than the median (48.9%) for *Pezizomycotina* (Storck 1966; Nishida 2015) and the average for *Ascomycota* (> 50%) (Li and Du 2014). High GC content has been indicated to play a role in complex environmental adaptation and horizontal gene transfer (Mann and Chen 2010) and is linked with halotolerance in prokaryotes (Jacob 2012). High GC content has also been linked with thermal stability of the DNA through base pair stacking (Yakovchuk et al. 2006), higher affinity of the histones (Nishida 2015), and lower occurrence of transposable elements (TEs) (Muszewska et al. 2017), while high AT content has been linked to anaerobic fungi (Wilken et al. 2020). This indicates that *E. atlantica* is adapted to an environment with high salt content or an environment with active exchange of genes, increasing the GC content, which in turn decreased the portion of TEs. The amount of SSRs were only about 1.16% in *E. atlantica*, 4.32% in *A. encephaloides* and 1.48% in *C. marina*. The fragmentation of the genomes makes it difficult to assess the amount of SSRs accurately.

Of the different gene clusters with characterized compounds that were detected in *E. atlantica*, only botrydial has not been described from *Acremonium* or *Emericellopsis*. The total number of BGCs detected by antiSMASH were 35 clusters, slightly lower than average for *Sordariomycetes* (Rokas et al. 2018; Robey et al. 2020). Isolates within *Emericellopsis* are capable of producing a range of NRPS-derived peptides (Cole and Rolinson 1961; Argoudelis et al. 1974; Ishiyama et al. 2000; Rogozhin et al. 2018; Baranova et al. 2019). This indicates that *E. atlantica* is a promising source of potentially novel NRPS produced peptides. However, application of the OSMAC (One Strain Many Compounds) approach in culturing or heterologous expression and gene-knockout experiments may be needed to produce these putatively novel NRPS-peptides and characterize the gene clusters (De Mattos-Shipley et al. 2018).

Comparison of terrestrial and marine fungal genomes are still in an early phase. A study of *Hypoxylaceae* revealed that two closely related species of different origin (terrestrial vs marine) showed a relatively low portion, 5.5%, of species-specific genes in the marine isolate (Wibberg et al. 2021). The authors hypothesized that these genes might be involved in osmotolerance and nutrient uptake. Few of these specific genes had characterized functions, and it is therefore difficult to assess the marine nature of isolates based on genomic information alone. In addition, epigenetic modification may play a large role in adaptations to different environments (Kronholm et al. 2016). Further comparison and characterizations of genes and genomes and their regulation are needed to understand the specific adaptations of marine fungi.

### Biosystematics and sexual reproduction of *Emericellopsis atlantica*

Morphologically, *E. atlantica* is differentiated from the other *Emericellopsis* species in the marine and alkaline clade by irregularly shaped guttules and longer phialides. The distinct morphology supported the phylogenetic placement on a separate branch within the marine clade of *Emericellopsis*, closely related to *E. pallida* and *E. phycophila* that are morphologically different. The major branches in the three clades of “terrestrial”, marine and “soda soil” *Emericellopsis* were supported in both Bayesian and maximum likelihood models. As Gonçalves et al. (2020) noted, the clades do not contain species with the same traits and isolation locality. *Emericellopsis cladophorae* and *E. enteromorphae* were isolated from algae in estuarine environments and were placed in the “alkaline soda soil” and “terrestrial” clade, respectively. The long branches of the terrestrial clade were likely induced by missing data in three to four of the six loci in terrestrial isolates (Wiens 2006; Darriba et al. 2016) and in the three newly described species in Gonçalves et al. (2020). Only one of the terrestrial sequences, *E. minima* CBS871.68, contained all six loci. The close relation of these species makes it difficult to establish proper phylogenetic relations without sequence data from several loci. The lack of sequence data, together with “*Acremonium*” species in each of the three clades, as well as the placement of the algae associated *E. cladophorae* and *E. enteromorphae* outside of the marine clade supports the necessity of a taxonomic revision of the genus (Gonçalves et al. 2020).

Despite the lack of sporocarps during isolation and culturing, *E. atlantica* contained a complete MAT1–1 mating locus (*sla2*, *MATa3, MATa2, MATa1*, *apn1* and *cox6a*) on scaffold_14 and pheromone sensing protein. This indicates that sexual reproduction and sporocarp formation could be possible in the species if MAT1–2 exists (Klix et al. 2010). However, one can ask the question whether sexual reproduction would take place in the sponge-host in deep-sea environment. It is possible that the fungus is present elsewhere in other marine substrates or habitats that could function as suitable places for sexual reproduction.

### A generalist fungus with ability to degrade marine biomass

The growth characterization showed that the preferred substrate of *E. atlantica* was 0.4MEA and sponge extract in seawater. The slowest growth occurred in media prepared with distilled water. *Emericellopsis atlantica* grew on the control media, which means it could also utilize agar alone as a nutrient source. The results shows that the fungus prefers saline conditions with complex nutrient sources, at least in axenic laboratory culture. A growth optimum temperature of above 20 °C is similar to what is observed in some other marine fungi (Philomena 1980; Lorenz and Molitoris 1992; Pang et al. 2011). *Emericellopsis atlantica* grows at 2 °C, which makes it a psychrotrophic fungus (Hassan et al. 2016; Wang et al. 2017). The adaptation of *E. atlantica* to the marine environment is further supported by the presence of CAZyme classes relating to utilization of marine polysaccharides such as fucose, carrageenan and laminarin. However, no modular sulfatases with a CAZyme domain as described in Helbert (2017) were predicted from the JGI annotation. A manual search using the SulfAtlas database (Barbeyron et al. 2016) revealed several CAZymes with low E score against putative sulfatases and sulfatase domains. However, they did not contain the conserved peptide pattern of the catalytic site. Many marine polysaccharides have attached sulfate groups and removal of those is necessary for utilization of the sugars (Schultz-Johansen et al. 2018; Kappelmann et al. 2019). *Emericellopsis atlantica* contained six putative sulfatases, without CAZyme domains, annotated by JGI in the genome, which is three times more than *A. encephaloides* and *C. marina*, but less than the terrestrial species (11 and 22 genes). *Emericellopsis atlantica* lacked genes for polyphenol oxidase, of which the activity has been observed in degradation assays in the terrestrial clade of *Emericellopsis* (Zuccaro et al. 2004; Grum-Grzhimaylo et al. 2013). Marine clade *Emericellopsis* spp. were unable to degrade polyphenols. The absence of polyphenol oxidase and the presence of fucosidase (GH29/95/141) is in line with the detected phenotype of the marine clade of *Emericellopsis* (Zuccaro et al. 2004). The absence of polyphenol oxidase indicates that *E. atlantica* does not degrade gallotannins and ellagitannins from terrestrial sources (Cammann et al. 1989; Salminen et al. 2002; Zuccaro et al. 2004). There are other types of tannins such as phlorotannins in brown algae and other enzymes may be required to degrade them (Jormalainen et al. 2003; Zuccaro et al. 2004). Loss of the ability to break down gallotannins and ellagitannins is likely a specialization to the available substrates in the marine environment (Zuccaro et al. 2004).

Generalists tend to have a higher amount of CAZymes than specialists do in order to utilize a wider range of substrates (Zhao et al. 2014). *Emericellopsis atlantica* showed a wider range of enzymatic classes than the other fungi in this study (176 different classes). Considering that *E. atlantica* did not have the highest number of genes, but still had the highest diversity of enzymatic classes indicates an adaptation to utilize a wide diversity of substrates. The ability to process any source of nutrients efficiently would be beneficial in a nutrient poor ocean environment (Turley 2000). Sponges are natural filters for organic matter such as marine snow and naturally concentrate the availability of different nutrients, therefore fungi may exploit this by living within the sponge and adapt specifically to that environment (Anteneh et al. 2019).

Polysaccharide lyases occurred in fewer numbers in terrestrial saprophytic and facultative parasitic fungi, where some even lacked PLs altogether (Soanes et al. 2008; Zhao et al. 2014). PLs have been shown to be related to breakdown of pectins from cell walls in marine diatoms and seagrasses (Desikachary and Dweltz 1961; Ovodova et al. 1968; Hehemann et al. 2017; Hobbs et al. 2019). *Emericellopsis atlantica* had almost twice the number of PLs (17) compared to the other fungi in the CAZyme analysis. Interestingly, the number of PLs was significantly higher than in *A. niger* which is used as an industrial producer of different CAZymes (Chettri et al. 2020). Furthermore, the relative number of CAZymes with secretory signal was also high (38%). This indicates that *E. atlantica* can break down cell remnants in marine snow or pectin rich substrates in marine sediments or within the sponge host (Smith et al. 1992).

The presence of DNA photolyases in the genome could indicate that the species is not specifically adapted to dark deep-sea environments (Núñez-Pons et al. 2018). Partial loss of photolyases has previously been reported in white-nose fungi from bats as an adaptation to darkness (Palmer et al. 2018). The host sponge *Stelletta normani* was collected from 1350 m depth, but the type specimen of the species was collected from 330 m depth in Southern Norway (Sollas 1880). Other sources report specimens collected from the twilight (dysphotic) zone with small amounts of light penetration (Murillo et al. 2012). The sponge occurrence is not restricted to the deep-sea environment, which makes it logical that the associated *E. atlantica* has not lost its photolyases and is not an obligate deep-sea dweller.

## CONCLUSION

*Emericellopsis atlantica* is the first genome sequenced *Emericellopsis* species and a distinct marine fungus showing adaptations to utilize a range of different substrates in the marine environment. The nature of the relationship to the host sponge cannot be determined based on this single isolate. A large portion of predicted genes have unknown or general function prediction only, which underlines the need to sequence more genomes for comparative genomic analyses to identify possible mechanisms of adaptations. The *E. atlantica* genome also contained several unknown NRPS clusters and enzymes that warrant future research and may be of biotechnological and industrial interest. The three genomes we have presented here will contribute to the increased number of available marine fungal genomes and shedding light on the characteristics of marine fungi.

## Supplementary Information

**Additional file 1 **: **Supplementary data 1.** Sequencing details and statistics regarding library and method for assembly for each species.

**Additional file 2 **: **Supplementary data 2.** Overview of fungal accessions used with type status, isolation source and model selection for phylogenetic analysis.

**Additional file 3 **: **Supplementary data 3.** Phylogenetic tree produced by PhyML webpage used as supporting data for Fig. 1.

**Additional file 4 **: **Supplementary data 4.** Overview of functional and enzymatic classes of genes using KEGG and KOG annotation.

**Additional file 5 **: **Supplementary data 5.** Overview of detected classes, numbers of each class, number of genes with secretion signal.

**Additional file 6 **: **Supplementary data 6.** Graphic output from synteny analysis of BGCs in *E. atlantica*.

**Additional file 7 **: **Supplementary data 7.** Bioactivity data of fractions produced from fermentation of *E. atlantica*.

## Data Availability

The trees generated and/or analyzed during the current study are available in the TreeBASE repository, http://purl.org/phylo/treebase/phylows/study/TB2:S27616. Genomes and annotations are submitted to NCBI/GenBank under the accessions: PRJNA571189 (*E. atlantica*), PRJNA347005 (*A. encephaloides*), PRJNA347008 (*C. marina*). They are also available at MycoCosm from the following links: *E. atlantica:* https://mycocosm.jgi.doe.gov/Emericellopsis_atlantica *A. encephaloides:* https://mycocosm.jgi.doe.gov/Amylocarpus_encephaloides *C. marina:* https://mycocosm.jgi.doe.gov/Calycina_marina All data generated or analyzed during this study are included in this published article, accessions and its supplementary data files. Raw data used in the study are available from the corresponding author on request.

## ABBREVIATIONS

1KFG: The 1000 fungal genomes
AA: Auxiliary activity
ASW: Artificial seawater
BGC: Biosynthetic gene cluster
CAZymes: Carbohydrate active enzymes
CBM: Carbohydrate binding module
CE: Carboxyl esterases
CEGMA: Core Eukaryotic Genes Mapping Approach
DEPC: Diethyl pyrocarbonate
DNA: Deoxyribonucleic acid
EC: Enzyme classification
GH: Glycoside hydrolase
GT: Glycosyl transferase
ITS: NrDNA internal transcribed spacer region
JGI: Joint Genome Institute
KEGG: Kyoto Encyclopedia of Genes and Genomes
KOG: Eukaryotic Orthologous Groups of proteins
MEA: Malt extract agar
NRPS: Non-ribosomal peptide synthase
OA: Oatmeal agar
OSMAC: One strain many compounds
PCR: Polymerase chain reaction
PDA: Potato dextrose agar
PKS: Polyketide synthase
PL: Polysaccharide lyase
RiPP: Ribosomally synthesized and post-translationally modified peptide
RNA: Ribonucleic acid
*rpb2*: Gene for the second largest subunit of RNA polymerase II
SSR: Short simple repeats
TE: Transposable elements
*tef1*: Transcription elongation factor 1 gene
*tub2*: Tubulin beta chain gene
