# Supplementary material for: Genomic characterization of three marine fungi, including Emericellopsis atlantica sp. nov. with signatures of a generalist lifestyle and marine biomass degradation
Source: IMA Fungus. 2021 Aug 9;12:21. doi: 10.1186/s43008-021-00072-0 (PMC8351168; doi:10.1186/s43008-021-00072-0)
Supplement: Supplementary file 1 — Additional file 1 : Supplementary data 1. Sequencing details and statistics regarding library and method for assembly for each species. [file 43008_2021_72_MOESM1_ESM.docx]

# Hagestad et al. 2021, Genomic characterization of three marine fungi, including Emericellopsis atlantica sp. nov. with signatures of a generalist lifestyle and marine biomass degradation

# Supplementary data 1 – sequencing and assembly statistics

Acremonium sp. TS7 - DNA
Fungal Minimal Draft Genome

**1. Project Information**proposal_title: 1000 Fungal Genomes
ap_name: *Acremonium* sp. TS7 Minimal Draft
analysis_proj_id: 1092651
analysis_task_id: 125242
seq_proj_id: 1092660
organism_name: *Acremonium* sp. TS7
 superkingdom: Eukaryota
 kingdom: Fungi
 phylum: *Ascomycota*
 class: None
 order: None
 family: None
 genus: *Acremonium*
 species: *Acremonium* sp. TS7
 strain: TS7
data:
 library: GANCG
 platform: Illumina
 protocol: Illumina Regular Fragment, 300bp, Tubes
 sequencer: NovaSeq

**2. Assembly Information**#fungal_main_scaffolds
n_scaffolds: 114
n_contigs: 162
scaf_bp: 27300990
contig_bp: 27298605
gap_pct: 0.009
scaf_N50: 14
scaf_L50: 758458
ctg_N50: 17
ctg_L50: 450535
scaf_max: 1473484
ctg_max: 1294182
scaf_n_gt50K: 58
scaf_pct_gt50K: 97.285
gc_avg: 0.5418
gc_std: 0.0705
ploidy: 1
 *total_seq_cov: 347.58
 *total_assem_cov: 225.61 std: 498.13
 *RNAseq_capture: 99.38 %
 *EST_capture: 98.72 %
 *CEGMA_capture: 99.34 % uniq: 44.54 %
 *ITS_capture: 100.00 % hit_bp/its_bp: 579/579

**#fungal_mito_scaffolds**n_scaffolds: 1
n_contigs: 1
scaf_bp: 25688
contig_bp: 25688
gap_pct: 0.0
scaf_N50: 1
scaf_L50: 25688
ctg_N50: 1
ctg_L50: 25688
scaf_max: 25688
ctg_max: 25688
scaf_n_gt50K: 0
scaf_pct_gt50K: 0.0
gc_avg: 0.2906
gc_std: 0.0000
ploidy: 1
bias_stddev_est: 0.04
gc_of_frag_reads_pct: 29.1
genome_cov_est_frag: 105
indel_rate_consensus: na
ambiguous_base_rate_consensus: na
snp_rate_consensus: na
snp_rate_uncorrected_fragment: na
genome_size_est_bp: 25587
est_genome_repeat_at_K25_scale: 0.0 %

**#lib usage**type lib_name lib_stats insert_mean insert_sd min_read_len mean_read_len max_read_len n_reads pct_used seq_cov n_pairs phys_cov
frag STD 2+/-60 300 60 50 149 150 21410 99.0 123.8 10443 134.8
jump sLMP 2828+/-258 3028 258 100 100 100 6422 100.0 25.0 2812 362.8
 *total_assem_cov: 148.80
 *total_frag_cov: 123.80
 *total_jump_cov: 25.00
 *EST_capture: 0.05 %
**#fungal_ribo_scaffolds**n_scaffolds: 1
n_contigs: 1
scaf_bp: 11830
contig_bp: 11830
gap_pct: 0.0
scaf_N50: 1
scaf_L50: 11830
ctg_N50: 1
ctg_L50: 11830
scaf_max: 11830
ctg_max: 11830
scaf_n_gt50K: 0
scaf_pct_gt50K: 0.0
gc_avg: 0.5340
gc_std: 0.0000
ploidy: 1
bias_stddev_est: 14.02
gc_of_frag_reads_pct: 53.8
genome_cov_est_frag: 105
indel_rate_consensus: na
ambiguous_base_rate_consensus: na
snp_rate_consensus: na
snp_rate_uncorrected_fragment: na
genome_size_est_bp: 11711
est_genome_repeat_at_K25_scale: 0.0 %

**#lib usage**type lib_name lib_stats insert_mean insert_sd min_read_len mean_read_len max_read_len n_reads pct_used seq_cov n_pairs phys_cov
frag STD 8+/-60 306 60 122 149 150 9776 99.3 123.1 4780 135.8
jump sLMP 2757+/-256 2957 256 100 100 100 2930 100.0 24.8 1465 506.6
 *total_assem_cov: 147.90
 *total_frag_cov: 123.10
 *total_jump_cov: 24.80
 *EST_capture: 0.10 %

**4. Methods**The draft genome of *Acremonium* sp. TS7 was generated at the DOE Joint Genome Institute (JGI) using Illumina technology. An Illumina Regular Fragment, 300bp, Tubes standard shotgun library was constructed and sequenced using the NovaSeq, which generated 64263580 raw reads totaling 9.7038 Gb. All raw Illumina sequence data was filtered for artifact/process contamination using the JGI QC pipeline (1) which produced 63288878 reads totaling 9.4894 Gb. An automated attempt was made to reassemble any potential organelle (mitochondrion) from the filtered reads and remove any organelle- matching reads with kmer matching against the resulting contigs with an in-house tool (2). An assembly of the target genome was generated using the resulting nonOrganelle reads with SPAdes v3.12.0 (3) using the following parameters [--phred-offset 33 --cov-cutoff auto -t 16 -m 115 –k 25,55,95 --careful ]. Similar methodology (2), employing the UNITE rDNA database (10), was used to reassemble the ribosomal DNA from the filtered reads.
Completeness of the euchromatic portion of the genome assembly was assessed by aligning assembled consensus RNA sequence data with bbtools version 38.31 bbmap.sh [k=13 maxindel=100000 customtag ordered nodisk] and bbest.sh [fraction=85] (7). This is a routine test to determine whether we are missing significant portions of the genome.

Acremonium sp. TS7 - RNA

=====================
 PROJECT INFORMATION
=====================
Scientific Program: Fungal
Organism Name: Acremonium sp. TS7
Principal Investigator: Joseph Spatafora
Proposal ID: 662
Sequencing Project ID: 1092661
Final Deliverable Project ID: 1092650
Proposal Name: 1000 Fungal Genomes
Sequencing Project Name: Acremonium sp. TS7 Transcriptome

=====================
 SAMPLE SUMMARY
=====================
1=libraryName 2=SampleId 3=rawReads 4=filteredReads 5=sampleName 6=conditionNumber 7=sequencerType 8=runType 9=fileUsed
CWYAN 101219 177841410 167239730 Acremonium_RNA # NovaSeq 2x151 12673.2.276395.TCTCCGAT-ATCGGAGA.filter-RNA.fastq.gz

=====================
 ASSEMBLY STATISTICS
=====================
number of contigs: 31863
N50: 6568
longest contig: 34613
number of total bases: 135398524
median length: 3202
shortest contig: 201
# of long contigs (>= 1000 bp): 25296
# of medium contigs (>= 500 bp and < 1000 bp): 2794
# of short contigs (< 500 bp): 3773
Accuracy - Fraction of contigs mapping to the genome: 31714/31863 = 99.53%
Accuracy - Fraction of contigs mapping to the genome with high accuracy (thresh = 95 % of contig): 30649/31714 = 96.64%

=====================
 PROCESSING METHODS
=====================
Library Creation
----------------
Stranded RNASeq library(s) were created and quantified by qPCR.
Sequencing was performed using an Illumina instrument (refer to Sample Summary Table for specifics per library)

Read preprocessing:
-------------------
Raw fastq file reads were filtered and trimmed using the JGI QC pipeline resulting in the filtered fastq file (*.filter-RNA.fastq.gz files). Using BBDuk [1], raw reads were evaluated for artifact sequence by kmer matching (kmer=25), allowing 1 mismatch and detected artifact was trimmed from the 3' end of the reads. RNA spike-in reads, PhiX reads and reads containing any Ns were removed. Quality trimming was performed using the phred trimming method set at Q6. Finally, following trimming, reads under the length threshold were removed (minimum length 25 bases or 1/3 of the original read length - whichever is longer).

De novo Assembly
----------------
Filtered fastq files were used as input for de novo assembly of RNA contigs. Reads were assembled into consensus sequences using Trinity (ver. 2.3.2) [2]. Trinity partitions the sequence data into many individual de Bruijn graphs, each representing the transcriptional complexity at at a given gene or locus, and then processes each graph independently to extract full-length splicing isoforms and to tease apart transcripts derived from paralogous genes. Trinity combines three independent software modules: Inchworm, Chrysalis, and Butterfly, applied sequentially to process large volumes of RNA-seq reads. Trinity was run with the --normalize_reads (In-silico normalization routine) and --jaccard_clip (Minimizing fusion transcripts derived from gene dense genomes) options.

=====================
 SEQUENCE QC NOTES
=====================
CWYAN - 2% rRNA, 166M genome mapped reads (99.32%)
===============================================================

# Calycina marina - DNA

*Calycina marina* TRa3180A
Fungal Minimal Draft Genome
Jasmyn Pangilinan, [jlpangilinan@Lbl.gov](mailto:jlpangilinan@Lbl.gov)
20190118

**1. Project Information**proposal_title: 1000 Fungal Genomes
ap_name: Calycina marina TRa3180A Minimal Draft
analysis_proj_id: 1081706
analysis_task_id: 116246
seq_proj_id: 1081727
pi_contact: Spatafora, Joseph; [spatafoj@science.oregonstate.edu](mailto:spatafoj@science.oregonstate.edu)
sample_contact: Rama, Teppo; [teppo.rama@uit.no](mailto:teppo.rama@uit.no)
sp_proj_contact: Rama, Teppo; [teppo.rama@uit.no](mailto:teppo.rama@uit.no)
organism_name: *Calycina marina* TRa3180A
 superkingdom: Eukaryota
 kingdom: Fungi
 phylum: *Ascomycota*
 class: *Leotiomycetes*
 order: *Helotiales*
 family: *Hyaloscyphaceae*
 genus: *Calycina*
 species: *Calycina marina*
 strain: TRa3180A
data:
 library: GANCC
 platform: Illumina
 protocol: Illumina Regular Fragment, 300bp, Tubes
 sequencer: NovaSeq

**2. Assembly Information**#fungal_main_scaffolds
n_scaffolds: 1318
n_contigs: 1371
scaf_bp: 34209217
contig_bp: 34205481
gap_pct: 0.011
scaf_N50: 173
scaf_L50: 54098
ctg_N50: 186
ctg_L50: 52969
scaf_max: 375165
ctg_max: 375165
scaf_n_gt50K: 198
scaf_pct_gt50K: 53.912
gc_avg: 0.4762
gc_std: 0.0185
ploidy: 1
 *total_seq_cov: 483.23
 *total_assem_cov: 185.26 std: 247.84
 *EST_capture: 95.43 %
 *CEGMA_capture: 99.34 % uniq: 46.94 %
 *ITS_capture: 100.00 % hit_bp/its_bp: 506/506

**#fungal_mito_scaffolds**n_scaffolds: 1
n_contigs: 1
scaf_bp: 33655
contig_bp: 33655
gap_pct: 0.0
scaf_N50: 1
scaf_L50: 33655
ctg_N50: 1
ctg_L50: 33655
scaf_max: 33655
ctg_max: 33655
scaf_n_gt50K: 0
scaf_pct_gt50K: 0.0
gc_avg: 0.3042
gc_std: 0.0000
ploidy: 1
bias_stddev_est: 0.0
gc_of_frag_reads_pct: 30.9
genome_cov_est_frag: 0
indel_rate_consensus: na
ambiguous_base_rate_consensus: na
snp_rate_consensus: na
snp_rate_uncorrected_fragment: na
genome_size_est_bp: 0
est_genome_repeat_at_K25_scale: 0.0 %

**#lib usage**type lib_name lib_stats insert_mean insert_sd min_read_len mean_read_len max_read_len n_reads pct_used seq_cov n_pairs phys_cov
frag STD -7+/-60 291 60 62 149 150 28104 97.6 122.4 13299 131.6
jump sLMP 2826+/-265 3026 265 100 100 100 8430 100.0 25.1 3830 370.0
 *total_assem_cov: 147.50
 *total_frag_cov: 122.40
 *total_jump_cov: 25.10
 *EST_capture: 0.07 %

**#fungal_ribo_scaffolds**n_scaffolds: 1
n_contigs: 1
scaf_bp: 8427
contig_bp: 8427
gap_pct: 0.0
scaf_N50: 1
scaf_L50: 8427
ctg_N50: 1
ctg_L50: 8427
scaf_max: 8427
ctg_max: 8427
scaf_n_gt50K: 0
scaf_pct_gt50K: 0.0
gc_avg: 0.5081
gc_std: 0.0000
ploidy: 1
bias_stddev_est: 0.0
gc_of_frag_reads_pct: 51.5
genome_cov_est_frag: 0
indel_rate_consensus: na
ambiguous_base_rate_consensus: na
snp_rate_consensus: na
snp_rate_uncorrected_fragment: na
genome_size_est_bp: 0
est_genome_repeat_at_K25_scale: 0.0 %

**#lib usage**type lib_name lib_stats insert_mean insert_sd min_read_len mean_read_len max_read_len n_reads pct_used seq_cov n_pairs phys_cov
frag STD 10+/-60 308 60 49 149 150 7124 98.9 126.0 3478 136.8
jump sLMP 2754+/-253 2954 253 100 100 100 2130 99.9 25.4 1063 542.3
 *total_assem_cov: 151.40
 *total_frag_cov: 126.00
 *total_jump_cov: 25.40
 *EST_capture: 0.05 %

**4. Methods** The draft genome of Calycina marina TRa3180A was generated at the DOE Joint Genome Institute (JGI) using Illumina technology. An Illumina Regular Fragment, 300bp, Tubes standard shotgun library was constructed and sequenced using the NovaSeq, which generated 110822414 raw reads totaling 16.7342 Gb. All raw Illumina sequence data was filtered for artifact/process contamination using the JGI QC pipeline (1) which produced 110250938 reads totaling 16.5308 Gb. An automated attempt was made to reassemble any potential organelle (mitochondrion) from the filtered reads and remove any organelle-matching reads with kmer matching against the resulting contigs with an in-house tool (2). An assembly of the target genome was generated using the resulting nonOrganelle reads with SPAdes v3.12.0 (3) using the following parameters [--phred-offset 33 --cov-cutoff auto -t 16 -m 115 –k 25,55,95 --careful ]. Similar methodology (2), employing the UNITE rDNA database (11), was used to reassemble the ribosomal DNA from the filtered reads.
 Completeness of the euchromatic portion of the genome assembly was assessed by aligning assembled consensus RNA sequence data with ESTmapper (sim4db release 1919) (7) at 90% identity and 85% coverage. This is a routine test to determine whether we are missing significant portions of the genome.

Calycina marina - RNA

De novo assembly of RNAseq data for 1081728 Calycina marina TRa3180A is now complete.

=====================
 PROJECT INFORMATION
=====================
Scientific Program: Fungal
Organism Name: Calycina marina TRa3180A
Principal Investigator: Joseph Spatafora
Proposal ID: 662
Sequencing Project ID: 1081728
Final Deliverable Project ID: 1081705
Proposal Name: 1000 Fungal Genomes
Sequencing Project Name: Calycina marina TRa3180A Transcriptome

=====================
 SAMPLE SUMMARY     
=====================
1=libraryName   2=SampleId      3=rawReads      4=filteredReads 5=sampleName    6=conditionNumber       7=sequencerType 8=runType       9=fileUsed
CWYAH   94303   130889650       129023082       Calycina_RNA    #       NovaSeq 2x151   12673.2.276395.CATCGTGA-TCACGATG.filter-RNA.fastq.gz

=====================
 ASSEMBLY STATISTICS
=====================
number of contigs: 43973
N50: 8015
longest contig: 15333
number of total bases: 86679537
median length:  1286
shortest contig:  201
# of long contigs (>= 1000 bp):  24486
# of medium contigs (>= 500 bp and < 1000 bp):  5802
# of short contigs (< 500 bp):  13685
Accuracy - Fraction of contigs mapping to the genome:  43590/43973 =  99.13%
Accuracy - Fraction of contigs mapping to the genome with high accuracy (thresh = 95 % of contig):  38603/43590 =  88.56%

=====================
 PROCESSING METHODS
=====================
Library Creation
----------------
Stranded RNASeq library(s) were created and quantified by qPCR.
Sequencing was performed using an Illumina instrument (refer to Sample Summary Table for specifics per library)

Read preprocessing:
-------------------
Raw fastq file reads were filtered and trimmed using the JGI QC pipeline resulting in the filtered fastq file (*.filter-RNA.fastq.gz files). Using BBDuk [1], raw reads were evaluated for artifact sequence by kmer matching (kmer=25), allowing 1 mismatch and detected artifact was trimmed from the 3' end of the reads.  RNA spike-in reads, PhiX reads and reads containing any Ns were removed. Quality trimming was performed using the phred trimming method set at Q6.  Finally, following trimming, reads under the length threshold were removed (minimum length 25 bases or 1/3 of the original read length - whichever is longer).

De novo Assembly
----------------
Filtered fastq files were used as input for de novo assembly of RNA contigs. Reads were assembled into consensus sequences using Trinity (ver. 2.3.2) [2]. Trinity partitions the sequence data into many individual de Bruijn graphs, each representing the transcriptional complexity at at a given gene or locus, and then processes each graph independently to extract full-length splicing isoforms and to tease apart transcripts derived from paralogous genes. Trinity combines three independent software modules: Inchworm, Chrysalis, and Butterfly, applied sequentially to process large volumes of RNA-seq reads. Trinity was run with the --normalize_reads (In-silico normalization routine) and --jaccard_clip (Minimizing fusion transcripts derived from gene dense genomes) options. For additional information see: <http://trinityrnaseq.github.io>

References
----------
1. BBDuk: <https://sourceforge.net/projects/bbmap/>
2. Trinity: Grabherr MG, Haas BJ, Yassour M, Levin JZ, Thompson DA, Amit I, Adiconis X, Fan L, Raychowdhury R, Zeng Q, Chen Z, Mauceli E, Hacohen N, Gnirke A, Rhind N, di Palma F, Birren BW, Nusbaum C, Lindblad-Toh K, Friedman N, Regev A. Full-length transcriptome assembly from RNA-seq data without a reference genome. Nat Biotechnol. 2011 May 15;29(7):644-52. doi: 10.1038/nbt.1883. PubMed PMID: 21572440.

=====================
 SEQUENCE QC NOTES
=====================
CWYAH - 1% rRNA, 127M genome mapped reads (98.69%)

===============================================================

# Amylocarpus encephaloides - DNA

Amylocarpus encephaloides TRa018bII
Fungal Minimal Draft Genome

**1. Project Information**proposal_title: 1000 Fungal Genomes
ap_name: Amylocarpus encephaloides TRa018bII Minimal Draft
analysis_proj_id: 1081698
analysis_task_id: 116240
seq_proj_id: 1081723
pi_contact: Spatafora, Joseph; [spatafoj@science.oregonstate.edu](mailto:spatafoj@science.oregonstate.edu)
sample_contact: Rama, Teppo; [teppo.rama@uit.no](mailto:teppo.rama@uit.no)
sp_proj_contact: Rama, Teppo; [teppo.rama@uit.no](mailto:teppo.rama@uit.no)
organism_name: Amylocarpus encephaloides TRa018bII
 superkingdom: Eukaryota
 kingdom: Fungi
 phylum: Ascomycota
 class: Leotiomycetes
 order: Helotiales
 family: None
 genus: Amylocarpus
 species: Amylocarpus encephaloides
 strain: TRa018bII
data:
 library: GANCB
 platform: Illumina
 protocol: Illumina Regular Fragment, 300bp, Tubes
 sequencer: NovaSeq

**2. Assembly Information**#fungal_main_scaffolds
n_scaffolds: 2381
n_contigs: 2509
scaf_bp: 46286790
contig_bp: 46279561
gap_pct: 0.016
scaf_N50: 168
scaf_L50: 74588
ctg_N50: 200
ctg_L50: 65840
scaf_max: 419988
ctg_max: 371100
scaf_n_gt50K: 278
scaf_pct_gt50K: 64.724
gc_avg: 0.4486
gc_std: 0.1040
ploidy: 1
 *total_seq_cov: 798.51
 *total_assem_cov: 127.83 std: 62.42
 *RNAseq_capture: 99.06 %
 *EST_capture: 97.38 %
 *CEGMA_capture: 99.56 % uniq: 44.32 %
 *ITS_capture: 100.00 % hit_bp/its_bp: 500/500

**#fungal_mito_scaffolds**n_scaffolds: 2
n_contigs: 2
scaf_bp: 56361
contig_bp: 56361
gap_pct: 0.0
scaf_N50: 1
scaf_L50: 48756
ctg_N50: 1
ctg_L50: 48756
scaf_max: 48756
ctg_max: 48756
scaf_n_gt50K: 0
scaf_pct_gt50K: 0.0
gc_avg: 0.3083
gc_std: 0.1403
ploidy: 1
bias_stddev_est: 0.21
gc_of_frag_reads_pct: 32.6
genome_cov_est_frag: 103
indel_rate_consensus: na
ambiguous_base_rate_consensus: na
snp_rate_consensus: na
snp_rate_uncorrected_fragment: na
genome_size_est_bp: 57308
est_genome_repeat_at_K25_scale: 4.0 %

**#lib usage**type lib_name lib_stats insert_mean insert_sd min_read_len mean_read_len max_read_len n_reads pct_used seq_cov n_pairs phys_cov
frag STD -6+/-60 292 60 51 149 150 47190 99.1 124.3 23212 132.0
jump sLMP 2848+/-254 3048 254 100 100 100 14128 99.9 25.1 6703 387.7
 *total_assem_cov: 149.40
 *total_frag_cov: 124.30
 *total_jump_cov: 25.10
 *EST_capture: 0.63 %

**#fungal_ribo_scaffolds**n_scaffolds: 1
n_contigs: 1
scaf_bp: 7605
contig_bp: 7605
gap_pct: 0.0
scaf_N50: 1
scaf_L50: 7605
ctg_N50: 1
ctg_L50: 7605
scaf_max: 7605
ctg_max: 7605
scaf_n_gt50K: 0
scaf_pct_gt50K: 0.0
gc_avg: 0.5507
gc_std: 0.0000
ploidy: 1
bias_stddev_est: 0.0
gc_of_frag_reads_pct: 55.9
genome_cov_est_frag: 0
indel_rate_consensus: na
ambiguous_base_rate_consensus: na
snp_rate_consensus: na
snp_rate_uncorrected_fragment: na
genome_size_est_bp: 0
est_genome_repeat_at_K25_scale: 0.0 %

**#lib usage**type lib_name lib_stats insert_mean insert_sd min_read_len mean_read_len max_read_len n_reads pct_used seq_cov n_pairs phys_cov
frag STD 10+/-60 306 60 52 148 150 6424 98.7 124.3 3145 131.1
jump sLMP 2823+/-262 3023 262 100 100 100 1732 100.0 22.8 866 627.7
 *total_assem_cov: 147.10
 *total_frag_cov: 124.30
 *total_jump_cov: 22.80
 *EST_capture: 0.55 %

**4. Methods** The draft genome of Amylocarpus encephaloides TRa018bII was generated at the DOE Joint Genome Institute (JGI) using Illumina technology. An Illumina Regular Fragment, 300bp, Tubes standard shotgun library was constructed and sequenced using the NovaSeq, which generated 248263736 raw reads totaling 37.4878 Gb. All raw Illumina sequence data was filtered for artifact/process contamination using the JGI QC pipeline (1) which produced 246556252 reads totaling 36.9605 Gb. An automated attempt was made to reassemble any potential organelle (mitochondrion) from the filtered reads and remove any organelle-matching reads with kmer matching against the resulting contigs with an in- house tool (2). An assembly of the target genome was generated using the resulting nonOrganelle reads with SPAdes v3.12.0 (3) using the following parameters [--phred-offset 33 --cov-cutoff auto –t 16 -m 115 -k 25,55,95 --careful ]. Similar methodology (2), employing the UNITE rDNA database (10), was used to reassemble the ribosomal DNA from the filtered reads.

 Completeness of the euchromatic portion of the genome assembly was assessed by aligning assembled consensus RNA sequence data with bbtools version 38.34 bbmap.sh [k=13 maxindel=100000 customtag ordered nodisk] and bbest.sh [fraction=85] (7). This is a routine test to determine whether we are missing significant portions of the genome.

 1) [genome.jgi.doe.gov/lookup?keyName=jgiProjectId&keyValue=1081697](http://genome.jgi.doe.gov/lookup?keyName=jgiProjectId&keyValue=1081697)

 2) James Han: organelleAssem.py; A 2 million read subsample is generated using bbtools version 38.34 reformat.sh (7) using "sampleseed=1". The subsampled data is additionally filtered for length and quality using bbtools version 38.34 reformat.sh (7), with options "qtrim=t trimq=5 minavgquality=5 minlength=101 maxns=3", and subsequently assembled together with Velvet version 2.1.7 (4) using velvetg "-cov_cutoff 20". The resulting assembly is aligned to the NCBI refseq.mitochondrion database with BLAST megablast version 2.2.26 (8) with a minimum percent identity of 80% to identify organelle. A secondary assembly is performed with Velvet version 2.1.7 (4) using cov_cutoff, max_coverage, and exp_cov cutoffs defined from the coverages associated with the contigs previously identified as organelle. Read pairs providing linking support between the assembled contigs are identified by aligning the original input fastq to a version of the assembled contigs with all bases masked with N with exception to the terminal 300 bases, with bwa version 0.7.4-r385 (9) using "mem -t 16". The linking read pairs are used in conjunction with NCBI alignment results to refseq.mitochondrion to identify trusted organelle contigs. Main genome 18S ribosomal elements are identified by alignment to NCBI nt database with BLAST megablast version 2.2.26 (8) with a minimum percent identity of 80% and excluded from the list. An enriched set of organelle reads is then created from the original input fastq reads by kmer matching with bbtools version 38.34 bbduk (7), using defaults, against the resulting white list of organelle contigs. Those that do not match the organelle contigs are output into a separate nonOrganelle fastq for downstream assembly. 125X of the enriched organelle matching read set is then coassembled together with 25X simulated 1000 +/- 50 bp insert long mate-pairs, generated from the organelle contigs with wgsim version 0.3.1-r13 (5) using "-d 1000 -s 50", with AllPathsLG release R46652 (6) to produce a final mitochondrion assembly.

 3) Bankevich et al. (2012) SPAdes: A New Genome Assembly Algorithm and Its Applications to Single-Cell Sequencing. Journal of Computational Biology 9(5): 455–477. doi: 10.1089/cmb.2012.0021.

 4) Zerbino and Birney. (2008) Velvet: algorithms for de novo short read assembly using de Bruin graphs. Genome Res 18(5) 821-829. doi: 10.1101/gr.074492.107.

 5) <https://github.com/lh3/wgsim>

 6) Gnerre et al. (2010) High-quality draft assemblies of mammalian genomes from massively parallel sequence data. Proc Natl Acad Sci 108:4 1513-1518. doi: 10.1073/pnas.1017351108.

 7) B. Bushnell: BBTools software package, <http://sourceforge.net/projects/bbmap>.

 8) <https://blast.ncbi.nlm.nih.gov/Blast.cgi>

 9) Li H. and Durbin R. (2009) Fast and accurate short read alignment with Burrows-Wheeler Transform. Bioinformatics 25:1754-60. doi: 10.1093/bioinformatics/btp324.

 10) Koljalg et al. (2013) Towards a unified paradigm for sequence-based identification of Fungi. Molecular Ecology, DOI: 10.1111/mec.12481.

For additional information please contact:
Alicia Clum, [aclum@lbl.gov](mailto:aclum@lbl.gov)
Bill Andreopoulos, [wandreopoulos@lbl.gov](mailto:wandreopoulos@lbl.gov)

This file was automatically generated by the fungal_min.spades software (version 2.0).

# Amylocarpus encephaloides - RNA

De novo assembly of RNAseq data for 1081724 Amylocarpus encephaloides TRa018bII is now complete.
=====================
 PROJECT INFORMATION
=====================
Scientific Program: Fungal
Organism Name: Amylocarpus encephaloides TRa018bII
Principal Investigator: Joseph Spatafora
Proposal ID: 662
Sequencing Project ID: 1081724
Final Deliverable Project ID: 1081697
Proposal Name: 1000 Fungal Genomes
Sequencing Project Name: Amylocarpus encephaloides TRa018bII Transcriptome

=====================
 SAMPLE SUMMARY
=====================
1=libraryName 2=SampleId 3=rawReads 4=filteredReads 5=sampleName 6=conditionNumber 7=sequencerType 8=runType 9=fileUsed
CWYAC 94299 124372854 98545840 Amylocarpus_RNA # NovaSeq 2x151 12673.2.276395.CTTCGTTC-GAACGAAG.filter-RNA.fastq.gz

=====================
 ASSEMBLY STATISTICS
=====================
number of contigs: 37966
N50: 7371
longest contig: 18543
number of total bases: 89464071
median length: 1705
shortest contig: 201
# of long contigs (>= 1000 bp): 24338
# of medium contigs (>= 500 bp and < 1000 bp): 5149
# of short contigs (< 500 bp): 8479
Accuracy - Fraction of contigs mapping to the genome: 37603/37966 = 99.04%
Accuracy - Fraction of contigs mapping to the genome with high accuracy (thresh = 95 % of contig): 35762/37603 = 95.10%

=====================
 PROCESSING METHODS
=====================
Library Creation
----------------
Stranded RNASeq library(s) were created and quantified by qPCR.
Sequencing was performed using an Illumina instrument (refer to Sample Summary Table for specifics per library)

Read preprocessing:
-------------------
Raw fastq file reads were filtered and trimmed using the JGI QC pipeline resulting in the filtered fastq file (*.filter-RNA.fastq.gz files). Using BBDuk [1], raw reads were evaluated for artifact sequence by kmer matching (kmer=25), allowing 1 mismatch and detected artifact was trimmed from the 3' end of the reads. RNA spike-in reads, PhiX reads and reads containing any Ns were removed. Quality trimming was performed using the phred trimming method set at Q6. Finally, following trimming, reads under the length threshold were removed (minimum length 25 bases or 1/3 of the original read length - whichever is longer).

De novo Assembly
----------------
Filtered fastq files were used as input for de novo assembly of RNA contigs. Reads were assembled into consensus sequences using Trinity (ver. 2.3.2) [2]. Trinity partitions the sequence data into many individual de Bruijn graphs, each representing the transcriptional complexity at at a given gene or locus, and then processes each graph independently to extract full-length splicing isoforms and to tease apart transcripts derived from paralogous genes. Trinity combines three independent software modules: Inchworm, Chrysalis, and Butterfly, applied sequentially to process large volumes of RNA-seq reads. Trinity was run with the --normalize_reads (In-silico normalization routine) and --jaccard_clip (Minimizing fusion transcripts derived from gene dense genomes) options. For additional information see: <http://trinityrnaseq.github.io>

References
1. BBDuk: <https://sourceforge.net/projects/bbmap/>
2. Trinity: Grabherr MG, Haas BJ, Yassour M, Levin JZ, Thompson DA, Amit I, Adiconis X, Fan L, Raychowdhury R, Zeng Q, Chen Z, Mauceli E, Hacohen N, Gnirke A, Rhind N, di Palma F, Birren BW, Nusbaum C, Lindblad-Toh K, Friedman N, Regev A. Full-length transcriptome assembly from RNA-seq data without a reference genome. Nat Biotechnol. 2011 May 15;29(7):644-52. doi: 10.1038/nbt.1883. PubMed PMID: 21572440.

=====================
 SEQUENCE QC NOTES
=====================
CWYAC - 11% rRNA, 98M genome mapped reads (99.06%) ===============================================================

# Supplementary data – sequencing (*mitochondrion*)

James Han: organelleAssem.py; A 2 million read subsample is generated using bbtools version 38.31 reformat.sh (Bushnell) using "sampleseed=1". The subsampled data is additionally filtered for length and quality using bbtools version 38.31 reformat.sh (Bushnell), with options "qtrim=t trimq=5 minavgquality=5 minlength=101 maxns=3", and subsequently assembled together with Velvet version 2.1.7 (Zerbino and Birney 2008) using velvetg "-cov_cutoff 20". The resulting assembly is aligned to the NCBI refseq.mitochondrion database with BLAST megablast version 2.2.26 (Morgulis and others 2008) with a minimum percent identity of 80% to identify organelle. A secondary assembly is performed with Velvet version 2.1.7 (Zerbino and Birney 2008) using cov_cutoff, max_coverage, and exp_cov cutoffs defined from the coverages associated with the contigs previously identified as organelle. Read pairs providing linking support between the assembled contigs are identified by aligning the original input fastq to a version of the assembled contigs with all bases masked with N with exception to the terminal 300 bases, with bwa version 0.7.4-r385 (Li and Durbin 2009) using "mem -t 16". The linking read pairs are used in conjunction with NCBI alignment results to refseq.mitochondrion to identify trusted organelle contigs. Main genome 18S ribosomal elements are identified by alignment to NCBI nt database with BLAST megablast version 2.2.26 (Morgulis and others 2008) with a minimum percent identity of 80% and excluded from the list. An enriched set of organelle reads is then created from the original input fastq reads by kmer matching with bbtools version 38.31 bbduk (Bushnell), using defaults, against the resulting white list of organelle contigs. Those that do not match the organelle contigs are output into a separate non-Organelle fastq for downstream assembly. 125X of the enriched organelle matching read set is then coassembled together with 25X simulated 1000 +/- 50 bp insert long mate-pairs, generated from the organelle contigs with wgsim version 0.3.1-r13 (Li 2011) using "-d 1000 -s 50", with AllPathsLG release R46652 (Gnerre and others 2011) to produce a final mitochondrion assembly.

Bushnell B. BBTools software package [Internet]. Available from: h
<ttp://sourceforge.net/projects/bbmap>

Gnerre S, MacCallum I, Przybylski D, Ribeiro FJ, Burton JN, Walker BJ, Sharpe T, Hall G, Shea TP, Sykes S et al. . 2011. High-quality draft assemblies of mammalian genomes from massively parallel sequence data. Proceedings of the National Academy of Sciences of the United States of America 108:1513-1518.

Li H. Wgsim [Internet]. Available from: <https://github.com/lh3/wgsim>

Li H, Durbin R. 2009. Fast and accurate short read alignment with Burrows-Wheeler transform. Bioinformatics 25:1754-1760.

Morgulis A, Coulouris G, Raytselis Y, Madden TL, Agarwala R, Schäffer AA. 2008. Database indexing for production MegaBLAST searches. In: editor^editors. Bioinformatics. ed.: Bioinformatics. p. 1757-1764.

Zerbino DR, Birney E. 2008. Velvet: Algorithms for de novo short read assembly using de Bruijn graphs. Genome Research 18(5):821-829.
