# Supplementary figures and images for: Genomic characterization of three marine fungi, including Emericellopsis atlantica sp. nov. with signatures of a generalist lifestyle and marine biomass degradation
Source: IMA Fungus. 2021 Aug 9;12:21. doi: 10.1186/s43008-021-00072-0 (PMC8351168; doi:10.1186/s43008-021-00072-0)

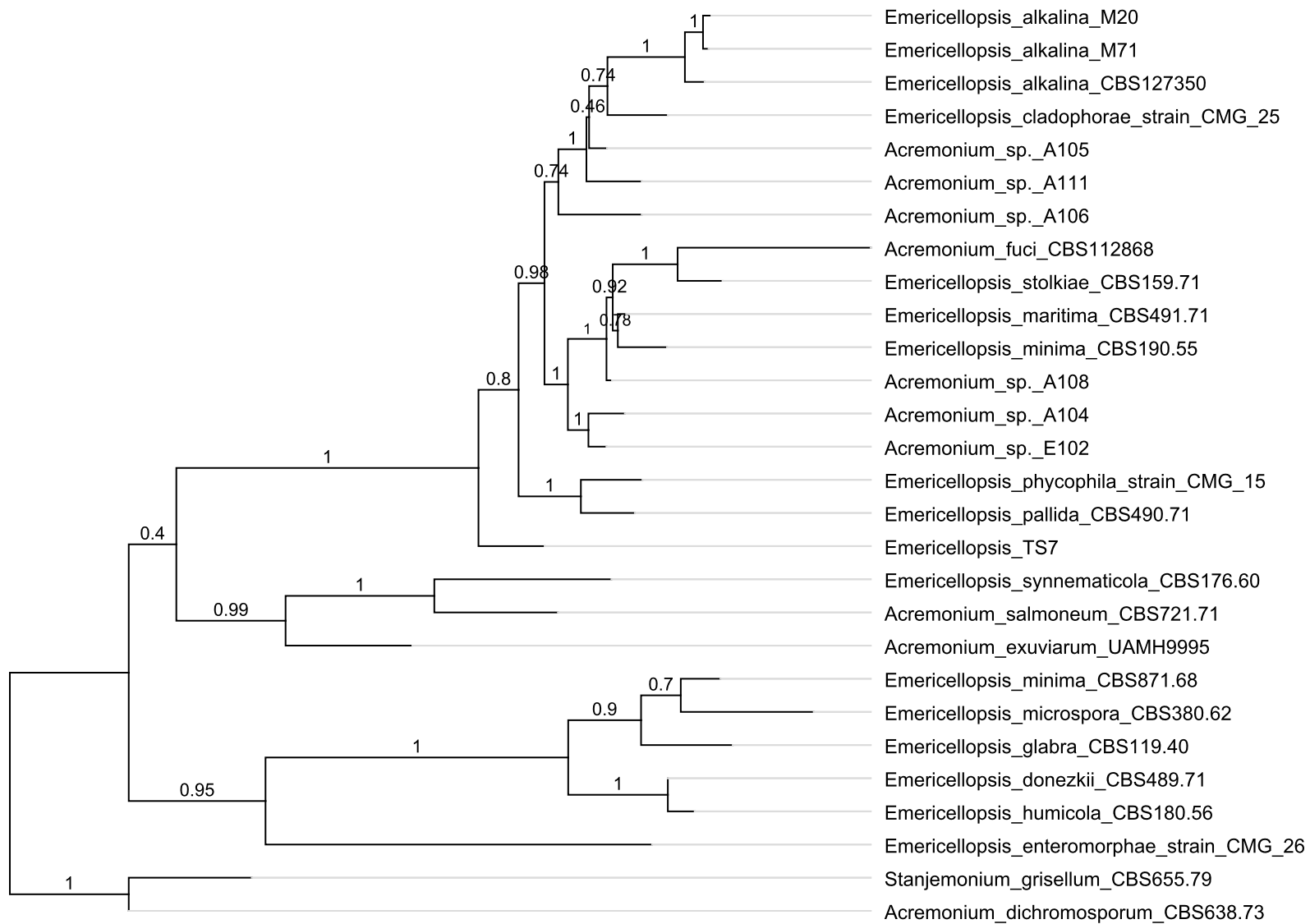

0.01

Supplement: Supplementary file 3 — Additional file 3 : Supplementary data 3. Phylogenetic tree produced by PhyML webpage used as supporting data for Fig. 1. [file 43008_2021_72_MOESM3_ESM.pdf]
