# Supplementary material for: Genomic characterization of three marine fungi, including Emericellopsis atlantica sp. nov. with signatures of a generalist lifestyle and marine biomass degradation
Source: IMA Fungus. 2021 Aug 9;12:21. doi: 10.1186/s43008-021-00072-0 (PMC8351168; doi:10.1186/s43008-021-00072-0)

Hagestad et al. 2021, Genomic characterization of three marine fungi, including Emericellopsis atlantica sp. nov. with signatures of a generalist lifestyle and marine biomass degradation

Supplementary data 6 – clinker synteny

Ascochlorine cluster


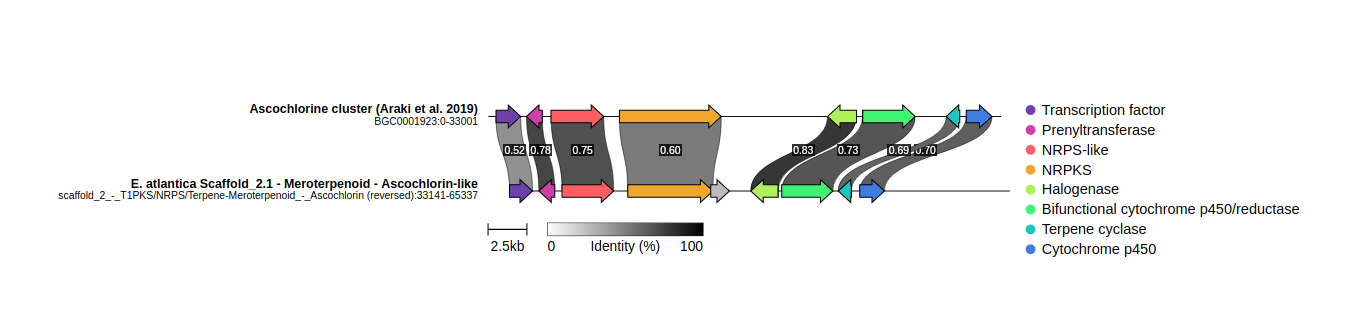


Leucinostatin cluster


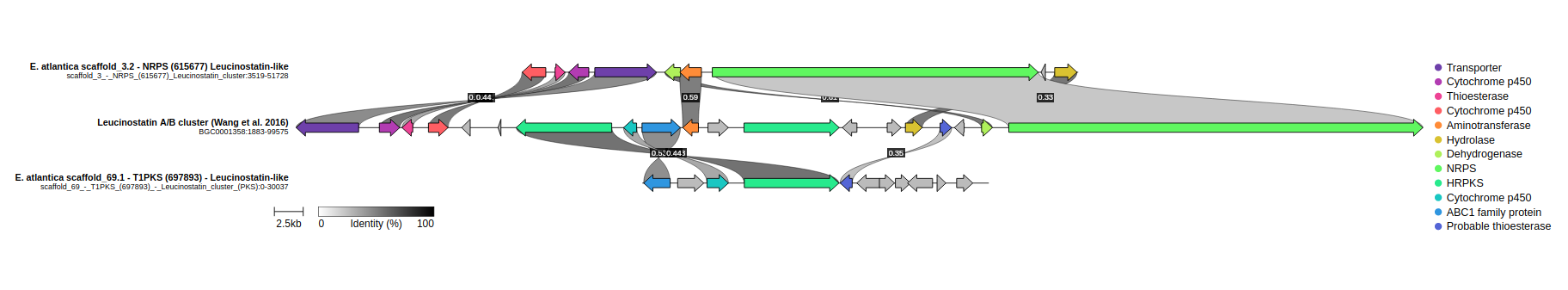


Botrydial cluster


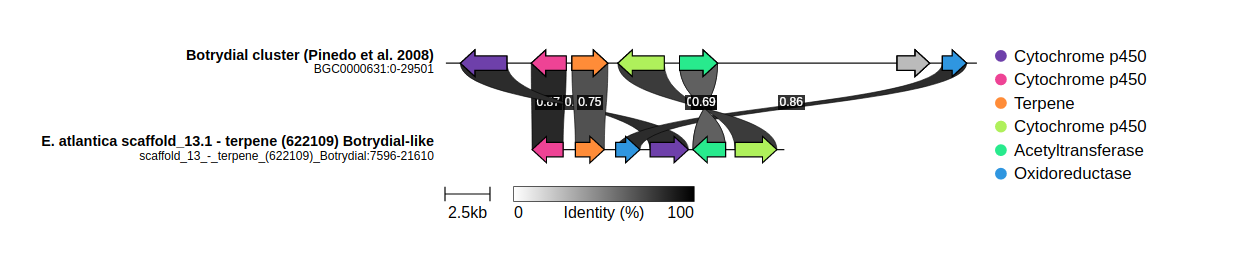


Cephalostatin cluster


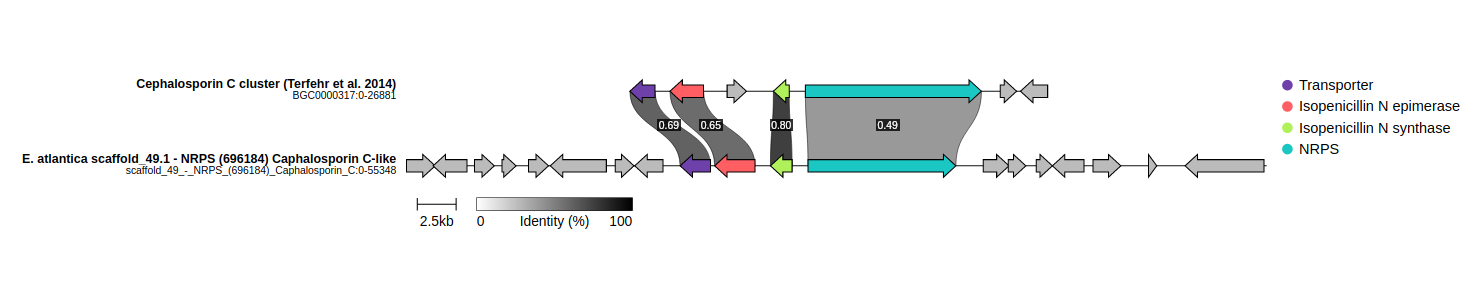


Helvolic acid cluster


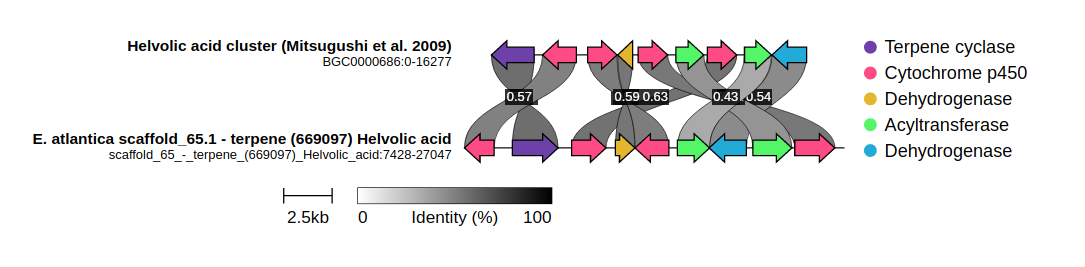

Supplement: Supplementary file 6 — Additional file 6 : Supplementary data 6. Graphic output from synteny analysis of BGCs in E. atlantica. [file 43008_2021_72_MOESM6_ESM.docx]
