# Supplementary material for: Genomic characterization of three marine fungi, including Emericellopsis atlantica sp. nov. with signatures of a generalist lifestyle and marine biomass degradation
Source: IMA Fungus. 2021 Aug 9;12:21. doi: 10.1186/s43008-021-00072-0 (PMC8351168; doi:10.1186/s43008-021-00072-0)
Supplement: Supplementary file 7 — Additional file 7 : Supplementary data 7. Bioactivity data of fractions produced from fermentation of E. atlantica. [file 43008_2021_72_MOESM7_ESM.docx]

# Hagestad et al. 2021, Genomic characterization of three marine fungi, including *Emericellopsis atlantica* sp. nov. with signatures of a generalist lifestyle and marine biomass degradation

# Supplementary data 7 – Bioactivity

## Bioactivity and dereplication of extracts from *Emericellopsis* TS7

*Emericellopsis* sp. TS7 cultures were grown for 3-6 weeks before being extracted using liquid-liquid or liquid-solid extraction with ethyl acetate, or SPE of liquid cultures with HP-20 resin followed by methanol elution of the extracted metabolites. The extracts were fractioned into eight fractions using flash chromatography. The bioactivity in the fractions from different cultures varied, indicating the likelihood of expression of potentially different active compounds. A total of 11 different extracts and eight different fractions from each extract (except F1, Verm and MWM) were tested. Fractions from fermentations in MPM-ASW, DMB and SAPM-ASW-media were active against biofilm formation (25 µg/mL) while fermentations in F1, AF, DPY, BRFT, Verm and MWM showed anti-bacterial activity to varying degree, Table 1. The highest antibacterial activity was observed in fermentations using AF and DPY. Fermentation in media containing both yeast extract and peptone such as DPY and AF showed activity against *E. faecium*. None of the fractions showed toxic activity against A2058 melanoma cells. Fractions from the DMB-fermentation displayed an immunostimulatory effect down to a level of 10 µg/mL in a dose dependent manner.

Table 1 - Bioactivity from the fractions of the *Emericellopsis* TS7 fermentations.

|  |  | **Frac. no.** | **Ext. met.** | **Cell prolif.** | **Immuno** | | **Biofilm inhibition** | | | **Growth inhib. assay** | | | | |
| --- | --- | --- | --- | --- | --- | --- | --- | --- | --- | --- | --- | --- | --- | --- |
| State | **Medium** |  |  |  | **IS** | **AIF** | **Form.** | **Estab.** | **Growth inh.** | **EF** | **EC** | **PA** | **SA** | **Str.B** |
| Liquid | **YES** | 5 | SPE | - | - | - | - | - | - | - | - | - | - | - |
| Liquid | **MPM** | 5 | SPE | - | - | - | 25 | - | - | - | - | - | - | - |
| Liquid | **DMB** | 5 | SPE | - | 10 | - | 25 | - | - | - | - | - | - | - |
| Liquid | **SAPM** | 5 | SPE | - | - | - | 25 | - | - | - | - | - | - | - |
| Liquid | **SDPM** | 5 | SPE | - | - | - | - | - | - | - | - | - | - | - |
| Liquid | **F1** | - | EtOAc | - |  |  | - | n.t. | - | - | - | - | - | 25 |
| Liquid | **AF** | 5 | EtOAc | - | - | - | - | - | 50 | 12.5 | - | - | - | 6.3 |
| Liquid | **DPY** | 5 | EtOAc | - | - | - | - | - | 50 | 25 | - | - | - | 6.3 |
| Solid | **BRFT** | 5 | EtOAc | - | - | - | - | - | - | - | - | - | - | - |
| Solid | **Verm** | - | EtOAc | - | n.t. | n.t. | - | - | 50 | - | - | - | - | 6.3 |
| Solid | **MWM** | - | EtOAc | - | n.t. | n.t. | - | - | - | - | - | - | - | 25 |

Frac. no. – Fraction number, Ext. met. – Extraction method, IS – Immunostimulatory, AIF – Anti-inflammatory, Form. – Biofilm formation, Estab. – Established biofilm, Growth inh. – Growth inhibition, EF – *Enterococcus faecium,* EC – *Escherichia coli*, PA – *Pseudomonas aeruginosa,* SA – *Staphylococcus aureus*, Str.B – *Streptococcus agalactiae*

Dereplication was performed using UHPLC-QToF-MS where the fractions were compared against each other and media controls. The second highest peak in the active fraction 5 from the AF and DPY fermentations was not present in the inactive fractions. The peak had a retention time of 7.45 min and a *m/z* of 567.2883 [M-H]^-^ in ESI- and a calculated elemental composition of C_33_H_43_O_8_ (calculated deprotonated monoisotopic mass: 567.2958 Da). A search in ChemSpider indicated that this compound could be helvolic acid (Fumigacin). To confirm the identity, we compared the endogenous compound with a commercial standard of helvolic acid (Sigma) on the UHPLC-QToF-MS. Both compounds had identical retention times, accurate mass and fragmentation pattern. The presence of the gene cluster of helvolic acid further supported the correct identification. The helvolic acid peak was followed by a smaller peak at m/z 569.3010 Da. This compound had a similar fragmentation pattern as helvolic acid (fragments were consistently 2 Da larger with few exceptions), and this was most likely 1,2-dihydrohelvolic acid.

The third most abundant peak in the fermentation using DMB had a *m/z* of 1050.7529 Da [M+H]^+^  with a calculated elemental composition of C_54_H_100_N_9_O_11_ (Calculated protonated monoisotopic mass: 1050.7542 Da). The fragmentation pattern indicated that the compound was a peptide, and a search in ChemSpider matched the elemental composition of the peptide acrepeptin C. However, the fragmentation pattern did not match with the amino acid composition of this peptide. Several other probable peptides (based on fragmentation) in the range of 800-1100 Da were also observed among the most abundant ions in the DMB sample, and these signals were not detected in the media control. However, none of the calculated elemental compositions matched known peptides in the ChemSpider database.

Methods:

## Cultivation for metabolite extraction, metabolite extraction and dereplication

The methods for extraction of metabolites, fractionation, UHPLC-HR-MS and dereplication have previously been described in Schneider et al. (2019).

### Extraction of metabolites

To prepare biomass for extraction of metabolites, mycelium of *Emericellopsis* TS7 was inoculated in 250 mL media in 1000 mL baffled culture flasks containing YES-ASW, MPM-ASW, DMB, SAPM-ASW, SDPM-ASW, F1, AF or DPY. Solid culture fermentations were performed with BRFT, Verm or MWM. Liquid fermentations of *Emericellopsis* TS7 in YES-ASW, MPM-ASW, DMB, SAPM-ASW and SDPM-ASW was extracted by solid phase extraction (SPE) using Diaion®HP-20 resin (Supelco Analytica, Bellefonte, PA, USA). The resin was activated before 40 g of resin was added to 1 L of culture 3-7 days before extraction. The resin was separated from the media and washed with MilliQ to remove remnants from the culture media. Methanol was used to elute metabolites from the resin. Extraction of metabolites from the resin was repeated once and the extract dried under reduced pressure at 40 °C. The dried extracts were dissolved in DMSO and stored at -20 °C.

Liquid-liquid and liquid-solid media extraction was performed using 1:1 ethyl acetate (EtOAc - Sigma) and liquid or solid fermentation culture. The solid culture was cut into pieces in the culturing flasks before addition of EtOAc. The fermentations of DPY, F1, AF, BRFT, Verm and MWM was extracted two times, and the organic phase was separated using a separation funnel or filter before the organic phase was evaporated to dryness under reduced pressure at 40 °C.

### Flash chromatography of extracts

Extracts from both liquid and solid cultures were fractioned into eight fractions using reversed phase flash liquid chromatography. The dried extracts were dissolved in 90% MeOH and 2 g of Diaion HP-20ss resin were added before it was dried under reduced pressure. Fractionation of the loaded resin was performed using a Biotage SP4 system with a stepwise gradient of MilliQ water to MeOH followed by MeOH:acetone (1:1) to 100% Acetone. The eight resulting fractions were dried under reduced pressure. Fractions were then dissolved in DMSO and stored at -20 °C.

### Mass spectrometry and dereplication

Fractions were diluted in methanol for analysis on an Acquity I-class UHPLC (Waters, Milford, MA, USA) coupled to a PDA detector and a Vion IMS QToF-MS (Waters). Separation of the samples was performed on an Acquity C-18 UPLC column (Waters). The separation gradient was 10-90 % MilliQ to acetonitrile (HiPerSolv, VWR) with 0.1 % formic acid (Sigma) during 12 min at a flow rate of 0.45 mL/min. Samples were run in ESI+ or ESI- mode, with the following settings: mass window 150-2000 Da, capillary voltage 0.8 kV, cone voltage 30 V, Source offset 50V, source temperature 120 °C, desolvation-gas N_2_, desolvation-gas temperature 350 °C, desolvation-gas flow 600 L/h, cone gas flow rate 50 L/h and analyzed using the software UNIFI v1.9.4 (Waters).

## Bioactivity

Bioactivity assays were performed as described in Lind et al. (2013), Lauritano et al. (2016) and Schneider et al. (2020).

### Growth inhibition assay

Briefly, the human pathogens *Staphylococcus aureus* (ATCC 25923), *Escherichia coli* (ATCC 259233) and *Pseudomonas aeruginosa* (ATCC 27853) from precultures in Muller Hinton Broth (MH, Becton, Dickinson and Company) and *Enterococcus faecalis* (ATCC 29122) and *Streptococcus agalactiae* (ATCC 12386) from precultures in Brain Heart Infusion Broth (BHI, Sigma-Aldrich) were grown to exponential growth phase, and a total of 1500-15000 CFU were added to each well of a 96-well plate (Nunclon, Thermo Scientific) with a total volume of 100 µL/well. Flash fractions were added to the wells with a final concentration of 100 µg/mL. The plates were incubated at 37 °C overnight before measuring the absorbance at 600 nm with a 1420 Multilabel Counter Victor^3^™ (Perkin Elmer, Waltham, MA, USA). Bacterial suspension diluted with MilliQ (1:1) acted as growth control, growth medium without bacteria as negative growth control. A dilution series of gentamycin was used as a positive assay control. Active fractions were tested at a serial dilution from 100 to 0.8 µg/mL.

### Biofilm inhibition assay

To test for biofilm formation inhibition, a Tryptic Soy Broth (TSB, Merck, Kenilworth, NJ, USA) preculture of *Staphylococcus epidermidis* (ATCC 35984) was diluted in media containing 1 % glucose before being transferred to a 96-well microtiter plate at 50 µL/well. Fifty microliters of each flash fraction were added to separate wells and incubated overnight. The bacteria were then removed and the plate washed in tap water. The biofilm was fixed before crystal violet was added to the wells followed by a 10 min incubation. Excess crystal violet solution was removed and the plate was dried, then 70 % EtOH was added and the plate incubated on a shaker for 10 min. To assess biofilm inhibition absorbance at 600 nm (OD600) was detected using a 1420 Multilabel Counter Victor3 plate reader. *S. haemolyticus* (clinical isolate 8-7A, University hospital, UNN, Tromsø, Norway), a non-biofilm forming clinical isolate, was used as a control and 50 µL TSB with Milli-Q (1:1) was used as media blank control. If the compounds tested inhibited bacterial growth such that the bacterial suspension was clear before removal, this was noted as bacterial inhibition rather than antibiofilm activity.

Removal of established biofilm was tested in a similar way as described above. *Staphylococcus epidermidis* (ATCC 35984) was incubated in TSB with 1 % glucose overnight at 37 °C. The next day the bacterial suspension was removed and washed carefully with PBS before the sample was added. After one day of incubation, the sample was removed and the biofilm was colored using crystal violet. The OD600 was measured using 1420 Multilabel Counter Victor3 plate reader. *S. haemolyticus* was used as a non-biofilm producing bacterial control.

### Cell proliferation assay

Toxicity of the fractions were assessed using a cell viability assay. Fractions were tested at 50 µg/mL final concentration in an MTS (3-(4,5-dimethylthiazol-2-yl)-5-(3-carboxymethoxyphenyl)-2-(4-sulfophenyl)-2H-tetrazolium) *in vitro* cell proliferation assay against A2058 human melanoma cancer cell line (ATCC CRL-11147). Final measurement of absorbance at 485 nm was performed on a DTX 880 multimode detector (Beckman Coulter) and cell viability was calculated. Growth media with and without 10 % DMSO was used as positive and negative control respectively.

### Anti-inflammatory and immunostimulatory assay

Anti-inflammatory: Prepared THP-1 cells (ATCC TIB-202) was seeded in 96-well plates at 10^6^ cells/ml and incubated for 48 h. The cells were checked and fresh media added, followed by 24 h incubation. Then, fresh media and 10 µL of sample (100 µg/mL final concentration) was added to each well. After 1 h of incubation 1 ng/mL of LPS (final concentration, Sigma) was added, followed by a 6 h incubation at 37 ºC. The reaction was stopped by freezing the plates at -80 °C immediately after incubation in preparation of Enzyme-linked immunosorbent assay (ELISA) assay. Positive and cell control is with and without LPS addition, respectively.

Immunostimulatory: This assay generally followed the exact same procedure as the anti-inflammatory assay, except after cell differentiation 90 µL fresh media and 10 µL of sample (100 µg/mL final concentration) was added to each well (omitting LPS addition). The reaction was stopped by freezing the plates at -80 °C immediately after incubation in preparation of ELISA. Positive and cell control is with and without LPS addition, respectively.

The secretion of TNFα was measured using ELISA. Between each of the following steps, the plates were washed with washing buffer (TBS with 0.05 % Tween-20, Sigma). All incubations were performed in room temperature in a shaking incubator. Samples were diluted 1:20 using assay diluent (Tris buffered saline - TBS with 1 % BSA) in the plate and a TNFα-standard dilution series was added to a prepared plate before incubation for 2 h. Biotin coupled anti-human antibody was added to each well followed by a 1 h incubation before diluted ExtrAvidin-Alkaline Phosphatease (Sigma) was added followed by 30 min incubation. Finally, 100 µL pNPP substrate (Sigma, 1 M dietanolamin, pH 9.8) was added to each well, incubated for 45 min and the plates were read in DTX 880 plate reader at 405 nm.
